# Adaptive resistance to PI3Kα-selective inhibitor CYH33 is mediated by genomic and transcriptomic alterations in ESCC cells

**DOI:** 10.1038/s41419-020-03370-4

**Published:** 2021-01-14

**Authors:** Yu-xiang Wang, Xu Zhang, Qing-yang Ma, Lan-dian Hu, Xi Zhang, Yi Wang, Lan Xu, Chun-hao Yang, Cun Tan, Xiang-yin Kong, Jian Ding, Ling-hua Meng

**Affiliations:** 1 Division of Anti-tumor Pharmacology, Shanghai Institute of Materia Medica, Chinese Academy of Sciences, 501 Haike Road, Shanghai, 201203 China; 2 University of Chinese Academy of Sciences, No. 19A Yuquan Road, Beijing, 100049 China; 3 Shanghai Institute of Nutrition and Health, Chinese Academy of Sciences, 320 Yueyang Road, Shanghai, 200031 China; 4 Department of Medicinal Chemistry, Shanghai Institute of Materia Medica, Chinese Academy of Sciences, 555 Zuchongzhi Road, Shanghai, 200120 China; 5 Division of Anti-tumor Pharmacology, State Key Laboratory of Drug Research, Shanghai Institute of Materia Medica, Chinese Academy of Sciences, 555 Zuchongzhi Road, Shanghai, 201203 China

**Keywords:** Cancer therapeutic resistance, Oncogenes

## Abstract

Phosphoinositide-3 kinase alpha-specific inhibitors (PI3Kαi) displayed promising potential for the treatment of esophageal squamous cell carcinoma (ESCC) with frequent activation in PI3K signaling. However, acquired resistance is likely to develop and limit the efficacy of PI3Kαi like other targeted therapies. To identify genomic adaptation to PI3Kαi, we applied whole-genome sequencing and detected gene mutation and amplification in four lines of ESCC cells established with adapted resistance to a novel PI3Kαi CYH33. Particularly, *HRAS*^*G12S*^ mutation was found in KYSE180C cells. Overexpression of HRAS^G12S^ in ESCC parental cells rendered resistance to CYH33. By contrast, down-regulation of HRAS^G12S^ restored the sensitivity of KYSE180C1 cells to CYH33, and combination of CYH33 and MEK162 displayed synergistic effect against KYSE180C1 cells and xenografts. Furthermore, elevated mTORC1, mitogen-activated protein kinase (MAPK), and c-Myc signaling pathways were found in resistant cells by RNA sequencing and combination of CYH33 and RAD001, MEK162, or OTX015 overcame the resistance to CYH33, which was accompanied with enhanced inhibition on S6, extracellular signal-regulated kinase 1 (ERK), or c-Myc, respectively. Overall, we characterized the adaptations to PI3Kαi in ESCC cells and identified combinatorial regimens that may circumvent resistance.

## Introduction

Esophageal cancer is the seventh most common cancer and the sixth leading cause of cancer-related mortality in the world, with an estimated 572,034 new cases diagnosed in 2018^1^. Esophageal squamous cell carcinoma (ESCC) and esophageal adenocarcinoma are the two major types of esophageal cancer, which tend to develop in different parts of the esophagus and are driven by different genetic alterations^2^. ESCC is the more common type of esophageal cancer on the global scale, especially in Eastern Asia as well as Eastern and Southern Africa, where about 90% of cases are ESCC^3^. The overall 5-year survival rate increased from 20.9% to 30.3% since 2003 to 2012 in China, and better outcome is associated with disease diagnosed at the early stage^4^. However, approximately 40% of patients with ESCC are diagnosed with advanced unresectable or metastatic disease. Definitive chemoradiotherapy (CRT) with 5-Fluorouracil (5-FU) and cisplatin is the standard treatment for patients with unresectable locally advanced ESCC^5^. Although clinical trials with antibodies against epidermal growth factor receptor (EGFR) or programmed cell death protein-1/programmed cell death-ligand 1 (PD-1/PD-L1) are in progress for the treatment of ESCC^6–8^, no molecularly targeted therapy has been approved up to date. ESCC remains a major unmet medical need worldwide.

To gain insight to the mechanism of pathogenesis, hundreds of ESCC samples have been profiled in a comprehensive manner using whole-exome sequencing (WES) and whole-genome sequencing (WGS) in the past 5 years^9–16^. Somatic copy number variations (CNV) involving 11q13.2-11q13.3 (47.2%), 3q26.32-3q26.33 (12.0%), and 9q21.3 (26.5%), as well as somatic mutations in tumor protein p53 (*TP53*, 80.5%), lysine methyltransferase 2D (*KMT2D*, 15.2%), notch receptor 1 (*NOTCH1*, 13.2%), nuclear factor erythroid 2 like 2 (*NFE2L2*, 9.3%), zinc finger protein 750 (*ZNF750*, 9.0%), FAT atypical cadherin 1 (*FAT1*, 8.8%), and phosphatidylinositol-4,5-bisphosphate 3-kinase catalytic subunit alpha (*PIK3CA*, 8.2%) genes have been frequently found in ESCC^17^. Notably, mutation of *PIK3CA* leads to hyper-activation of PI3Kα, which plays a key role in the regulation of multiple cellular events, including cell growth and proliferation^18^. Aberrant activation of PI3K also occurs frequently in ESCC via amplification of *PIK3CA*, hyper-activation of upstream receptor tyrosine kinases (RTKs), alteration in downstream effector *AKT*, as well as functional loss of phosphatase and tensin homolog (*PTEN*)^13^. Selective targeting PI3Kα has emerged as a promising approach for treatment of ESCC. Alpelisib, a PI3Kα-specific inhibitor, is currently in clinical trials to treat ESCC patients (NCT03292250 and NCT01822613)^19^. However, in the first-in-human clinical trial of alpelisib in solid tumors (NCT01387321), all the responding patients with head and neck squamous cell carcinomas (HNSCC) eventually became refractory to the treatment^20^. Given the similarities in terms of genetic characteristics between HNSCC and ESCC^21^, we would anticipate that efficacy of PI3Kα-specific inhibitors will be limited by the development of acquired resistance. It has been reported that activation of pim-1 proto-oncogene (PIM)^22^, p110β^23^, pyruvate dehydrogenase kinase 1 (PDK1)-serum/glucocorticoid-regulated kinase 1 (SGK1)^24^, and estrogen receptor^25^ mediated the resistance to PI3K inhibitors in breast cancer. However, the mechanism conferring resistance to PI3Kα-specific inhibitors in ESCC remains largely unknown.

CYH33 is a novel PI3Kα-selective inhibitor with a distinctive structure, which is discovered by our group and is currently in clinical trials for the treatment of solid tumors including advanced ESCC (NCT03544905)^26,27^. Our previous study has reported that CYH33 displayed significant activity against the proliferation of ESCC cells and the growth of xenografts derived from ESCC cells or ESCC patients^28^. CYH33 also sensitized ESCC to radiation by abrogating survival signals in tumor cells and tumor microenvironment^28^, indicating its potential in ESCC treatment. In this study, we established CYH33-resistant cell lines by incubating ESCC cells with progressive concentrations of CYH33. Mutation and gain of copy number were frequently found in CYH33-resistant cells, which were accompanied with activation of pathways involving mammalian target of Rapamycin complex1 (mTORC1), c-Myc, or mitogen-activated protein kinase (MAPK). Thus, inhibiting these pathways by RAD001, OTX015, or MEK162 sensitized the resistant ESCC cells to CYH33.

## Materials and methods

### Compounds

CYH33 was provided by Shanghai HaiHe Pharmaceutical Co. Ltd. Alpelisib, GSK21118436, MEK162, GDC0994, BI-D1870, RAD001, and OTX015 were purchased from Selleck Chemicals (Houston, TX, USA). For in vitro experiments, all compounds were dissolved in dimethyl sulfoxide (DMSO, Sigma, St. Louis, MO, USA) at the concentrations of 10 mM and stored at −20 ^o^C. For in vivo studies, CYH33 and MEK162 were solved in normal saline containing 0.5% Tween 80 (v/v; Sangon Biotech, Shanghai, China), and 1% CMC-Na (m/v; SINOPHARM, Beijing, China).

### Cell lines and cell culture

The esophageal squamous cell carcinoma COLO680N, KYSE30, KYSE180, KYSE70, KYSE140, KYSE150, KYSE410, KYSE450, and KYSE510 cells were kindly provided by Dr. Hideaki Shimada (Department of Surgery, Toho University School of Medicine). All cell lines were authenticated by analyzing short-tandem repeats (STR) by Genesky Biotechnologies Inc. (Shanghai, China). Cells were maintained in RPMI 1640 supplemented with 10% fetal bovine serum in a humidified atmosphere of 95% air and 5% CO_2_ at 37 °C. We established CYH33-resistant cells by exposing ESCC KYSE70, KYSE180, KYSE410, and KYSE510 cells to increasing concentrations of CYH33 as described previously^29^.

### Cell proliferation assay

Cell proliferation was evaluated using the sulforhodamine B (SRB, Sigma, ST, USA) assay as described previously^30^. The inhibitory rate was calculated using the formula: (OD_control cells_ − OD_treated cells_)/OD_control cells_ × 100% or (OD_control cells_ − OD_treated cells_)/(OD_control cells_ − OD_Day0 cells_) × 100%. IC_50_ or GI_50_ values were calculated by four parameter concentration response curve fitting with SoftMaxPro (Molecular Devices, California, USA).

### Next-generation sequencing

Genomic DNA and RNA were isolated and purified using AllPrep DNA/RNA mini Kit (Qiagen, Dusseldorf, Germany). For genomic DNA, paired-end libraries were constructed with insert size of approximately 400 bp using TruSeq Nano DNA Library Prep Kit (Illumina, California, USA). The constructed libraries were sequenced with the Illumina HiSeq X Ten sequencing system. Sequence reads were mapped onto hg19 reference genome with BWA (v0.7.9a). The alignment results were used to call SNV with GATK pipeline (v3.4). To determine copy number variations, we used CNVkit (v0.8.3). RNA-Seq libraries were constructed using paired-end adapters with an Illumina mRNA sequencing kit. The libraries were sequenced with the Illumina HiSeq X Ten sequencing system. Transcript quantification and differential expression analysis were performed using Salmon (v0.8.0) and DESeq2, respectively.

### Western blotting

Cell lysates were prepared and standard western blotting was performed with antibodies against HRAS (Abcam, Cambridge, UK), Akt, phospho-Akt (Ser473), phospho-Akt (Thr308), S6K, phospho-S6K (Thr389), 4EBP1, phospho-4EBP1 (Thr37/46), MEK, phospho-MEK (Ser217/221), extracellular signal-regulated kinase 1 (ERK), phospho-ERK (Thr202/Tyr204), p90RSK, phospho-p90RSK (Thr359/Ser363), c-Myc, S6, phospho-S6 (Ser235/236), phospho-S6 (Ser240/244) (Cell Signaling Technology, Danvers, MA, USA), and GAPDH, β-Actin (Sigma, St. Louis, MO, USA). Images were captured with the ImageQuant LAS 4000 system (GE, Boston, USA).

### Flow cytometry

Samples for analysis of cell cycle distribution were prepared as previously described^30,31^. Data were collected with a FACSCalibur Instrument (BD Biosciences, Franklin Lake, NJ, USA) and analyzed with FlowJo software.

### siRNA transfection

siRNA duplexes were synthesized by GenePharma (Shanghai, China). The sequences of siRNAs targeting HRAS were as follows: 5′-GCUGAUCCAGAACCAUUUUTT-3′, 5′-GAGGGCUUCCUGUGUGUGUTT-3′, 5′-UGCACGCACUGUGGAAUAUTT-3′. The negative control sequence was: 5′-UUCUCCGAACGUGUCACGUTT-3′. Cells seeded in 6-well plate were transfected with siRNAs using Lipofectamine RNAiMAX (Invitrogen, Carisbad, CA, USA) as described previously^32^. Cells were subjected to the following experiments 24 h post transfection.

### Plasmids and transfection

*HRAS* and *HRAS*^*G12S*^ genes were constructed into PGMLV-6395 vector, namely PGMLV-HRAS and PGMLV-HRAS^G12S^ by Genomeditech (Shanghai, China). HEK293T cells seeded in 6-well plates were transfected with PGMLV-6395, PGMLV-HRAS, or PGMLV-HRAS^G12S^ along with pCMV-VSV-G (#8454, Addgene) and pCMV-dR8.2 dvpr (#8455, Addgene) using Lipofectamine 2000 (Invitrogen, Carisbad, CA, USA) following the manufacturer’s instructions. Cell media containing viruses were collected 48 h after infection and filtered through a 0.45 μM filter. ESCC cells were infected with viruses in the presence of 6 μg/mL polybrene (Sigma, St. Louis, MO, USA). Cells stably expressing HRAS and HRAS^G12S^ were selected in the presence of 3 μg/mL puromycin.

### Combination analysis

Cells were treated with single agent alone or in combination, and inhibition on cell proliferation was determined by SRB assay. Combinatorial effect was analyzed by CalcuSyn software (Biosoft, Cambridge, UK) to determine the combination index (CI) using the median-effect method of Chou–Talalay^33^. A CI = 1 indicated an additive effect, a CI > 1 indicated antagonism, and a CI < 1 indicated synergism.

### Sanger sequencing

Total DNA was collected using the Qiagen DNeasy Blood/Tissue Kit according to the manufacturer’s instructions. Nested PCR was performed to generate target gene and the PCR products were sequenced by Sangon Biotech (Shanghai, China).

### Animal studies

All experiments were carried out according to the Institutional Ethical Guidelines on Animal Care and were approved by the Institute of Animal Care and Use Committee at Shanghai Institute of Materia Medica. For the experiment, 4–5-week-old male BALB/c athymic nude mice were obtained from the Shanghai Institute of Materia Medica (Shanghai, China). Then, 5 × 10^6^ KYSE180C1 or KYSE180-H cells suspended in Matrigel were injected subcutaneously into the right side of axillary. Tumor tissues were harvested and cut into small pieces with the size around 40 mm^3^ when the volume reached about 1000 mm^3^. The tumor pieces were then planted subcutaneously into BALB/c athymic nude mice. Animals were randomized to receive vehicle control or tested compounds. Mice were then administered orally with vehicle (normal saline containing 0.5% Tween 80 and 1% CMC-Na), CYH33 (10 mg/kg), MEK162 (5 mg/kg), or concurrently with CYH33 and MEK162 for 21 or 17 days. Body weight was recorded by electronic balance and the tumor volume was measured using microcalipers twice per week. The tumor volume (*V*) was calculated using the formula *V* = *a*^2^*b*/2, and *a* and *b* represented the tumor’s width and length, respectively.

### Statistical analysis

All data were represented as the mean and standard deviation (mean + SD) of at least three independent experiments unless otherwise stated in the figure legend. Data sets were tested for normality using the Shapiro-Wilk test. Statistical analyses were performed using Prism 8 (GraphPad, La Jolla, CA, USA). Statistical comparison was carried out with one-way ANOVA followed by Tukey multiple group comparison test among more than two groups. *P < 0.05, **P < 0.01, ***P < 0.001.

## Results

### ESCC cells developed acquired resistance after continuous exposure to PI3Kα inhibition

**Fig. 1:**
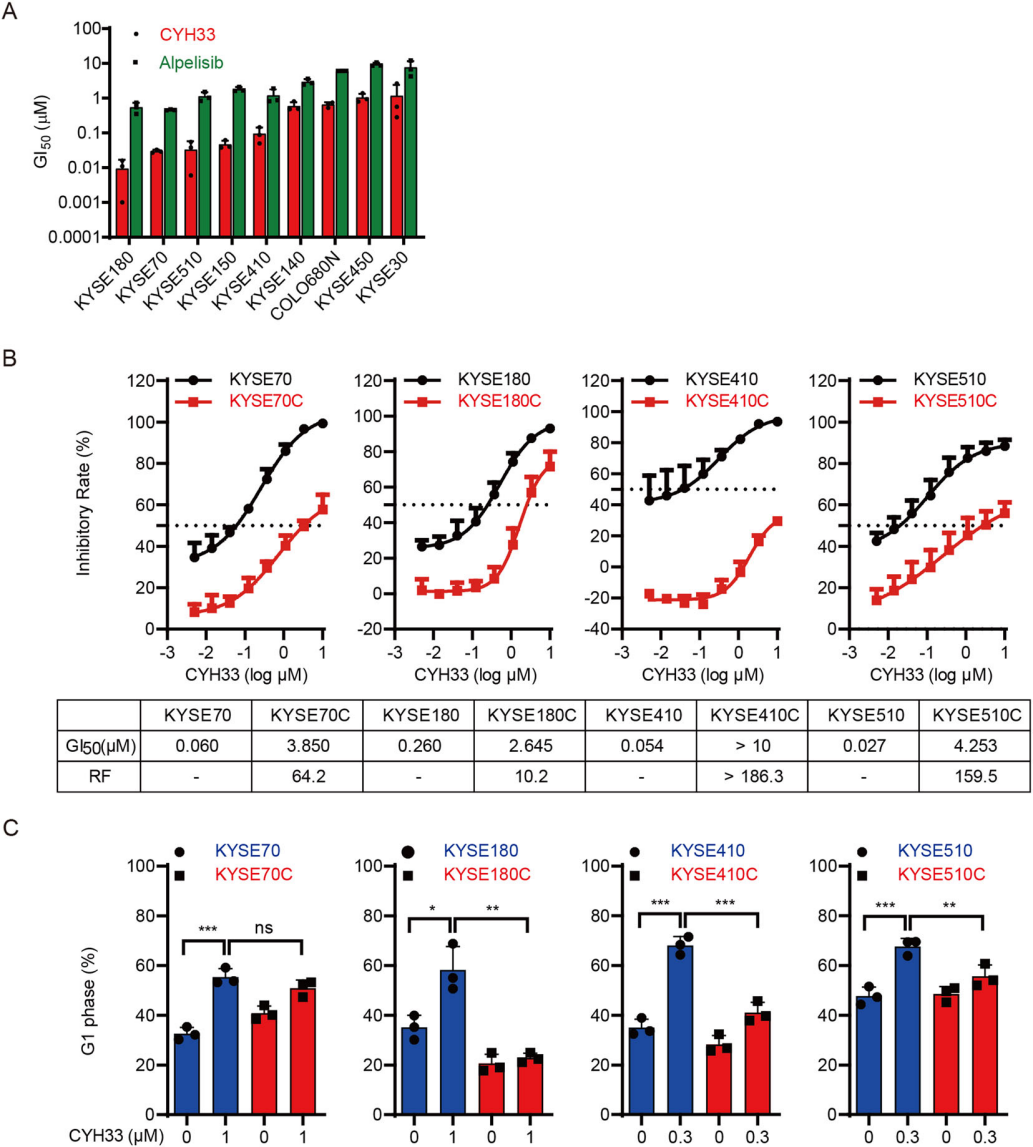
ESCC cells developed acquired resistance after continuous exposure to PI3Kα inhibition. **A** A series of ESCC cells were treated with CYH33 or alpelisib for 72 h and the GI_50_ values were determined with SRB assay (*n* = 3). **B** Parental and resistant cells were incubated with CYH33 for 72 h and cell proliferation was measured with SRB assay. Resistance factor (RF) was calculated by the ratio of GI_50_ obtained in CYH33-resistant cells and that in parental cells (*n* = 3). **C** Parental and resistant cells were treated with CYH33 at the indicated concentrations for 72 h and cell cycle distribution was analyzed by flow cytometry (*n* = 3). Data are presented as mean + SD. Differences between indicated groups were analyzed using one-way ANOVA with Tukey multiple group comparison test.*P < 0.05, **P < 0.01, ***P < 0.001.

We have reported that CYH33 is a novel PI3Kα-selective inhibitor and sensitize ESCC to radiation^28^. As PI3Kα is frequently hyper-activated in ESCC^13^, we investigated the anti-proliferative activity of CYH33 in a panel of ESCC cell lines. As shown in Fig. 1A, CYH33 significantly inhibited the proliferation of ESCC cells tested, with half-maximal growth inhibitory concentration (GI_50_) values ranging from 0.009 μM to 1.164 μM. Alpelisib, a well-known PI3Kα-selective inhibitor, displayed similar profile against the panel of cell lines but with less potency. Like other molecularly targeted inhibitors, we would anticipate that efficacy of PI3Kα-specific inhibitors against ESCC would be limited by the development of acquired resistance. To monitor the occurrence of resistance to PI3Kα inhibitors in ESCC and develop strategies to overcome the acquired resistance, we exposed 4 types of ESCC cell lines (KYSE70, KYSE180, KYSE410, and KYSE510) that are sensitive to PI3Kα inhibitors to increasing concentrations of CYH33 for about 6 months. These cells developed resistance to CYH33 and were named KYSE70C, KYSE180C, KYSE410C, and KYSE510C, respectively. As shown in Fig. 1B, the growth inhibitory curves of CYH33 in KYSE70C, KYSE180C, KYSE410C, and KYSE510C cells indicated that these cells were resistant to CYH33 compared with their parental cells. Accordingly, GI_50_ values of CYH33 obtained in CYH33-resistant cells were much higher than those obtained in parental cells, with the resistance factors more than 10 (Fig. 1B). Similar pattern was observed when these panel of cells were treated with alpelisib (Supplemental Fig. 1A), indicating that CYH33-resistant ESCC cells were cross-resistant to alpelisib. As PI3Kα inhibitors execute their anti-proliferative activity via arresting cells at G1 phase, we investigated whether the reduced sensitivity to CYH33 were due to different effects on cell cycle distribution in CYH33-resistant and parental cells. CYH33 and alpelisib significantly arrested cells at G1 phase in parental cells, while they displayed little effect on cell cycle distribution in resistant cells (Fig. 1C and Supplemental Fig. 1B, C). Thus, KYSE70C, KYSE180C, KYSE410C, and KYSE510C cells with adaptive resistance to CYH33 were successfully established.

### Gained mutations and amplification of chromosome segments were detected in CYH33-resistant cells

**Fig. 2:**
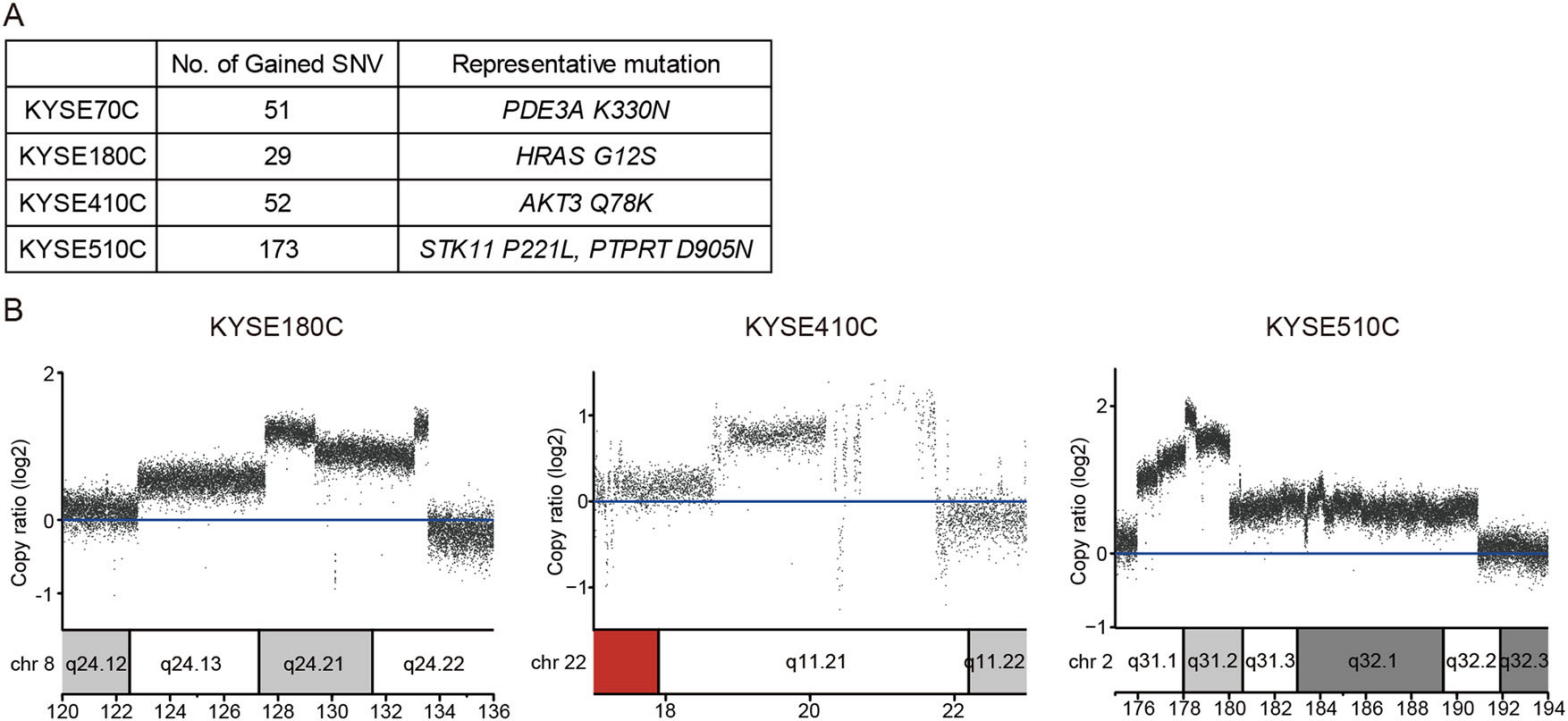
Gained mutations and amplification of chromosome segments were detected in CYH33-resistant cells. Genomic DNA purified from KYSE70C, KYSE180C, KYSE410C, and KYSE510C cells and their parental cells was subjected to whole-genome sequencing. **A** Number of non-synonymous mutations and representative mutations in KYSE70C, KYSE180C, KYSE410C, and KYSE510C cells compared to their parental counterparts. **B** Amplified chromosome segments in KYSE180C, KYSE410C, and KYSE510C cells compared to parental cells.

To elucidate the underlying mechanism of the acquired resistance to CYH33 in ESCC cells, we applied whole-genome sequencing and analyzed genomic alterations in CYH33-resistant cells compared to corresponding parental cells (PRJNA682323). DNA was purified from 4 types of CYH33-resistant cells as well as matched parental cells. The genomic DNA was captured and then sequenced using an Illumina Hiseq X system. The average distinct coverage of each base in the targeted region was 30-fold, and 95% of targeted bases were represented by at least 10 reads. Using stringent criteria for the analysis of these data, we identified 51, 29, 52, and 173 high-confidence non-synonymous somatic mutations in KYSE70C, KYSE180C, KYSE410C, and KYSE510C cells, respectively (Supplemental Tables S1–S4). However, mutation in *PIK3CA* was not identified in resistant cells. The representative oncogenic mutations were listed in Fig. 2A including *PDE3A*^*K330N*^ in KYSE70C cells, *HRAS*^*G12S*^ in KYSE180C cells, *AKT3*^*Q78K*^ in KYSE410C cells, and *STK11*^*P221L*^ and *PTPRT*^*D905N*^ in KYSE510C cells. In addition to gained mutations, we also detected amplification of multiple chromosome segments in the CYH33-resistant cells, such as amplification of chr8q24 in KYSE180C cells, chr22q11 in KYSE410C cells, and chr2q31 in KYSE510C cells (Fig. 2B). Amplified DNA segments localizing oncogenes such as activating transcription factor 2 (*ATF2*)^34^, TTN antisense RNA 1 (*TTN-AS1*)^35^, zinc fingers and homeoboxes 2 (*ZHX2*)^36^, Derlin-1 (*DERL1*)^37^, mediator complex subunit 15 (*MED15*)^38^, and MYC proto-oncogene (*MYC*)^39^ (Supplemental Tables S5–S7), which have been reported to mediate drug resistance in multiple cancer types. These results suggested that gained mutations and amplification of chromosome segments induced by CYH33 exposure might mediate resistance to the treatment.

### Acquired resistance to PI3Kα inhibition in ESCC cells was associated with hyper-activation of mTORC1, MAPK and c-Myc

**Fig. 3:**
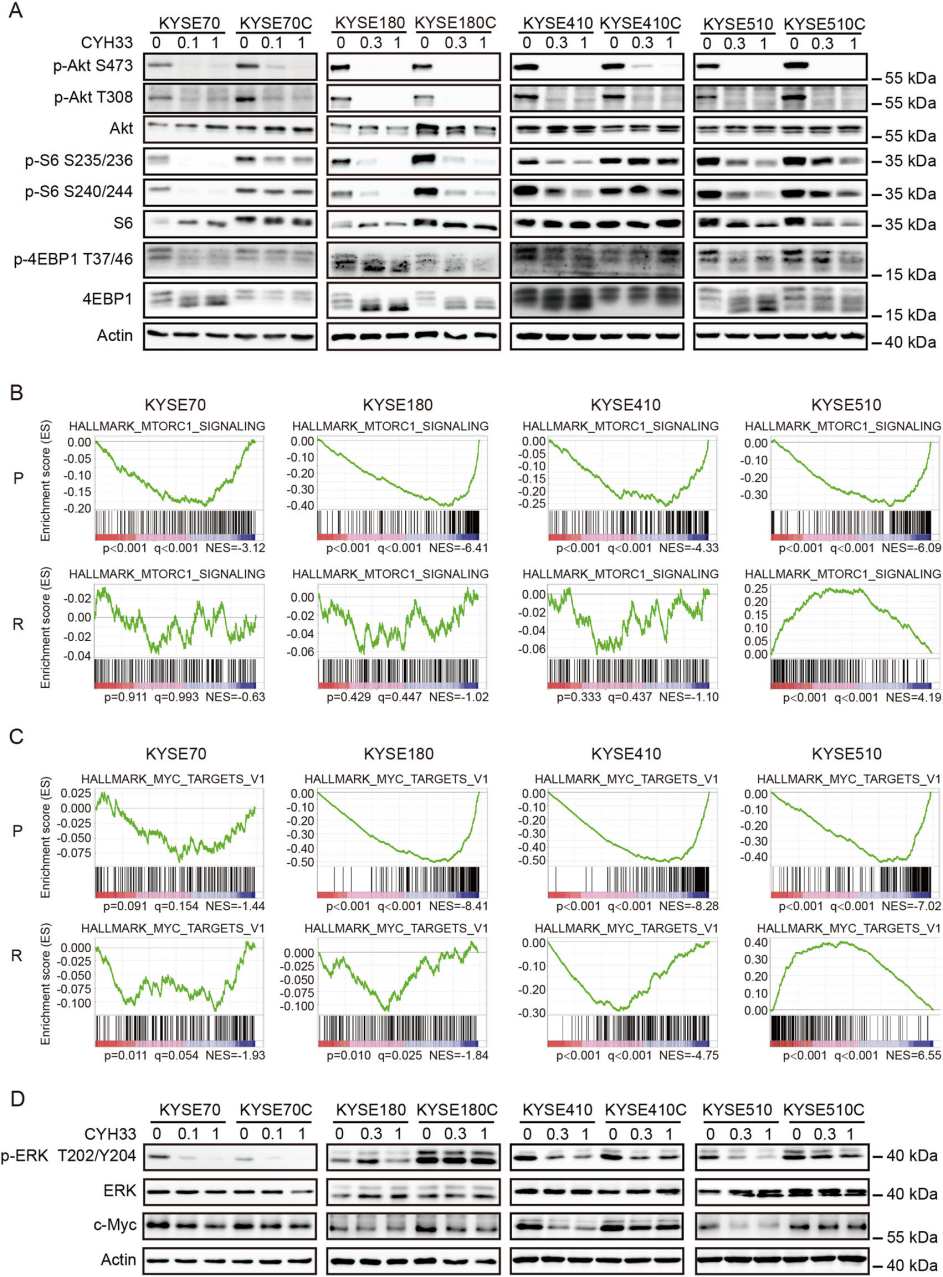
Acquired resistance to PI3Kα inhibition in ESCC cells was associated with hyper-activation of mTORC1, MAPK, and c-Myc. **A** Parental and resistant cells were treated with CYH33 at the indicated concentrations for 1 h. Cell lysates were subjected to western blotting with indicated antibodies. **B**, **C** CYH33-resistant and parental cells were treated with 1 μM CYH33 or DMSO for 24 h and RNA was purified and subjected to RNA sequencing. GSEA enrichment plot of differentially expressed genes in the gene set of mTORC1 signaling pathway (HALLMARK_MTORC1_SIGNALING) (**B**) and MYC targets (HALLMARK_MYC_TARGETS_V1) (**C**) upon CYH33 treatment in CYH33-resistant cells and parental cells. **D** CYH33-resistant cells and parental cells were treated with CYH33 at indicated concentrations for 1 h. Cell lysates were subjected to western blotting with indicated antibodies. Data shown are from three replicates or three independent experiments.

In order to identify mechanisms of the acquired resistance to CYH33, we interrogated the PI3K signaling upon CYH33 treatment in both resistant and parental cells. As shown in Fig. 3A, CYH33 suppressed the phosphorylation level of Akt, which is the intermediate downstream effector of PI3K, in resistant and parental cells in a dose-dependent manner, suggesting that the acquired resistance was not due to the lack of inhibition on its cellular target. We next checked the effect of CYH33 on the activity of mTORC1, which sits downstream of Akt. CYH33 slightly suppressed the level of phosphorylated ribosomal protein S6 (S6), a downstream effector of mTORC1, in KTSE70C, KYSE410C, and KYSE510C cells, while the phosphorylation of S6 was almost completely blocked by CYH33 in parental counterparts. These results indicated that mTORC1 remained activated independent of PI3Kα in cells resistant to CYH33.

To further investigate the mechanism mediating resistance to CYH33 in ESCC cells, global gene expression was profiled with RNA-Seq in CYH33-resistant cells as well as their parental cells (GSE162658). Gene enrichment analysis revealed that RAS signaling pathway, PI3K-Akt signaling pathway, and MAPK signaling pathway were highly activated in most CYH33-resistant cells (Supplemental Fig. 2A). To analyze the difference of gene expression profiles between CYH33-resistant cells and their matched parental cells upon CYH33 treatment, cells were treated with CYH33 at 1 μM for 24 h and RNA-seq was performed. Gene Set Enrichment Analysis (GSEA, http://software.broadinstitute.org/gsea/index.jsp) was then carried out (GSE162658). CYH33 significantly inhibited mTORC1 signaling pathway in all 4 types of parental cells, while CYH33 displayed little effect on this set of genes in KYSE70C, KYSE180C, and KYSE410C cells. mTORC1 signaling pathway even elevated in KYSE510C cells after CYH33 treatment (Fig. 3B). This finding was consistent with the results that mTORC1 remained activated independent of PI3Kα in cells resistant to CYH33 (Fig. 3A).

Similarly, “MYC_targets” gene set was down-regulated in CYH33-treated group in all 4 types of parental cells, while this effect was mild in KYSE70C, KYSE180C, and KYSE410C cells. “MYC targets” gene set was even up-regulated in KYSE510C cells treated with CYH33 (Fig. 3C). As stability of c-Myc protein is regulated by Ras-dependent phosphorylation^40^, we further detected MAPK pathway and c-Myc protein in CYH33-resistant cells and parental cells. ERK was hyper-activated in KYSE180C and KYSE510C cells compared to their parental cells, which was not attenuated by CYH33. Higher expression level of c-Myc was detected in KYSE180C cells than that in their parental cells. Although the level of c-Myc was similar in CYH33-resistant and parental cells, CYH33 markedly down-regulated c-Myc in KYSE410 and KYSE510 cells but not in KYSE410C and KYSE510C cells (Fig. 3D). These results demonstrated that mTORC1, c-Myc, and MAPK pathways were frequently activated independent of PI3Kα in CYH33-resistant ESCC cells.

### Gain-of-function mutation in *HRAS* rendered resistance to CYH33 in ESCC cells

**Fig. 4:**
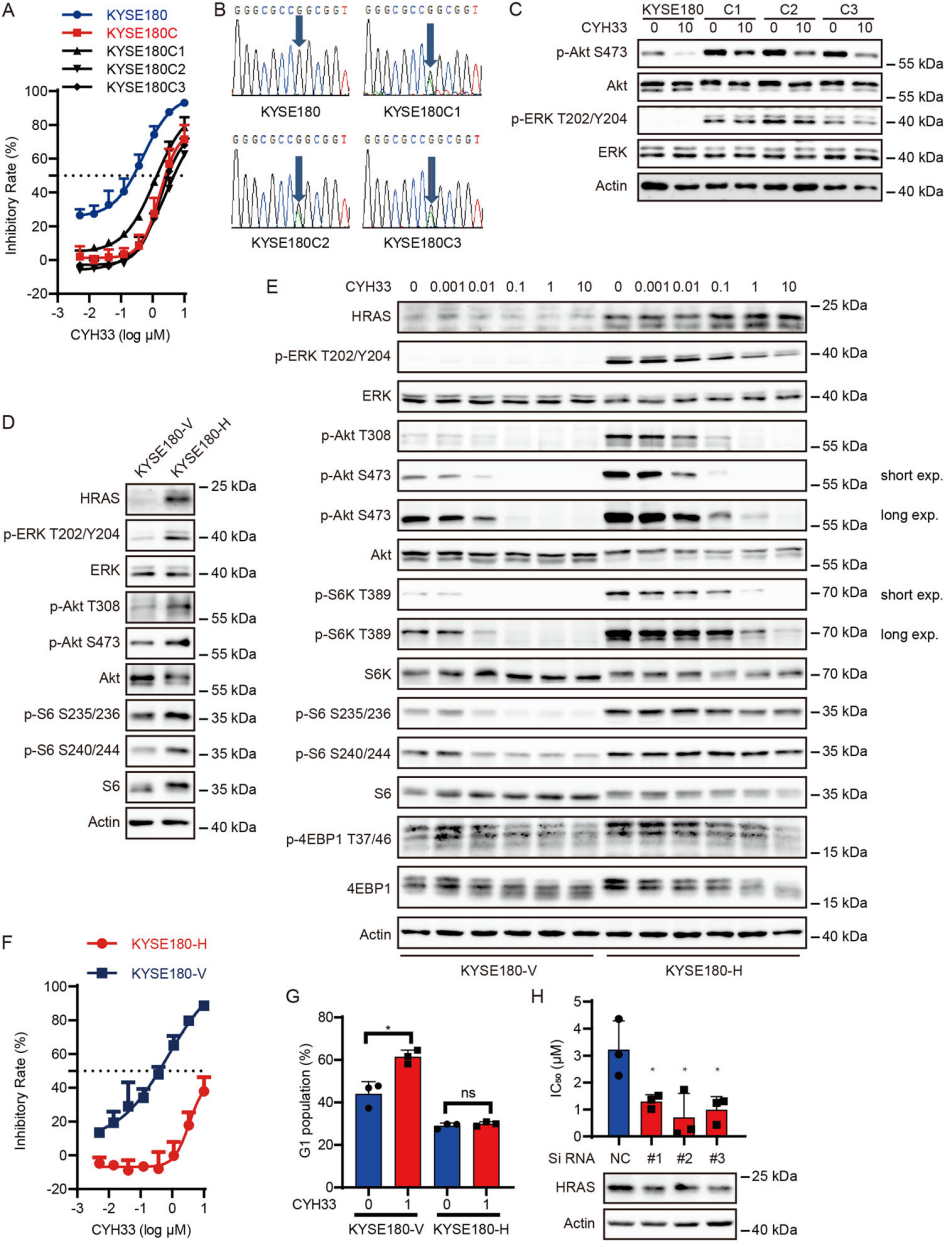
Gain-of-function mutation in *HRAS* rendered resistance to CYH33 in ESCC cells. **A** KYSE180, KYSE180C, and KYSE180C monoclonal cells were incubated with CYH33 for 72 h and cell proliferation was measured with SRB assay (*n* = 3). **B** Sanger sequencing of *HRAS* showing heterozygous mutation of *HRAS* at codon 12 in KYSE180C monoclonal cells. **C** KYSE180 and KYSE180C monoclonal cells were treated with CYH33 at 10 μM for 1 h, and cell lysates were subjected to western blotting with indicated antibodies. **D** KYSE180 cells were transfected with plasmid expressing HRAS^G12S^ mutant (KYSE180-H) or a vehicle plasmid (KYSE180-V), and cell lysates were subjected to western blotting with indicated antibodies. **E** KYSE180-V and KYSE180-H cells were treated with CYH33 at indicated concentrations for 1 h, and cell lysates were subjected to western blotting with indicated antibodies. **F** KYSE180-V and KYSE180-H cells were treated with CYH33 for 72 h and cell proliferation was measured with SRB assay (*n* = 3). **G** KYSE180-V and KYSE180-H cells were incubated with CYH33 for 24 h and cell cycle distribution was analyzed by flow cytometry (*n* = 3). **H** KYSE180C1 cells transfected with siRNAs targeting HRAS (#1, #2, and #3) or negative control siRNA (NC) were treated with CYH33 for 72 h and cell proliferation was measured with SRB assay (*n* = 3). Data shown are mean + SD or representative from three independent experiments. Differences between indicated groups were analyzed using one-way ANOVA with Tukey multiple group comparison test. *P < 0.05.

As mTOR and c-Myc have been reported to confer resistance to PI3K inhibitors in multiple cancers^41–43^ and we detected *HRAS*^*G12S*^ mutant in KYSE180C cells, we further investigated whether *HRAS*^*G12S*^ mutant was associated with resistance to CYH33. To verify mutation of *HRAS*^*G12S*^ in KYSE180C cells, we generated 3 lines of monoclonal cells from KYSE180C cells, namely KYSE180C1, KYSE180C2 and KYSE180C3. These monoclonal cells displayed similar resistance to CYH33 as KYSE180C cells (Fig. 4A). We amplified the DNA fragment containing *HRAS* codon 12 from the 3 lines of monoclonal cells by nested PCR. Sanger sequencing of the PCR products revealed a double peak at *HRAS* codon 12 (Fig. 4B), demonstrating a heterozygous G to A mutation in all KYSE180C monoclonal cells, which resulted in a heterozygous G12S mutation in *HRAS*. Accordingly, both Akt and ERK were hyper-phosphorylated in KYSE180C monoclonal cells compared to parental cells (Fig. 4C). Though CYH33 was able to inhibit the phosphorylation of Akt in KYSE180C monoclonal cells, phosphorylation of ERK remained unchanged upon CYH33 treatment (Fig. 4C). To determine whether ectopic expression of HRAS^G12S^ in ESCC cells is able to induce resistance to CYH33, we established KYSE180 cells stably overexpressing HRAS^G12S^ (KYSE180-H). Introduction of HRAS^G12S^ significantly activated PI3K pathway and MAPK pathway with enhanced level of phosphorylated Akt, ERK and S6 compared to those in KYSE180 cells transfected with a vehicle plasmid (KYSE180-V, Fig. 4D). Although CYH33 significantly blocked Akt phosphorylation in KYSE180-H cells, the phosphorylation of ERK, ribosomal protein S6 kinase 1 (S6K1) and S6 remained largely insensitive to CYH33 treatment in KYSE180-H cells (Fig. 4E). Accordingly, ectopic expression of HRAS^G12S^ significantly induced resistance to CYH33 and alpelisib in KYSE180 cells (Fig. 4F and Supplemental Fig. 3A), which was accompanied with reduction in cell population arrested at G1 phase after CYH33 treatment (Fig. 4G and Supplemental Fig. 3B). Overexpression of wild-type HRAS (180-WT) also rendered KYSE180 cells less sensitive to CYH33 (Supplemental Fig. 3C). However, cells expressing HRAS^G12S^ were more resistant to CYH33 compared to 180-WT cells though the expression of HRAS^G12S^ was lower than wild-type HRAS (Supplemental Fig. 3C). To investigate whether *HRAS*^*G12S*^ mutation was able to mediate resistance to CYH33 in other ESCC cells, we generated KYSE70, KYSE410, and KYSE510 cells expressing HRAS^G12S^, and found that these cells displayed reduced sensitivity to CYH33 (Supplemental Fig. 3D). To determine if HRAS^G12S^ activation is causal to CYH33 resistance, we employed HRAS-specific siRNAs to down-regulate its expression. As shown in Fig. 4H, knockdown of HRAS^G12S^ by siRNA sensitized KYSE180C1 cells to CYH33 demonstrated as reduced IC_50_ values of CYH33 against cell proliferation. Collectively, activation of HRAS by G12S mutation rendered resistance to CYH33 in ESCC cells.

### Inhibition of MAPK signaling pathway sensitized KYSE180C cells to CYH33

**Fig. 5:**
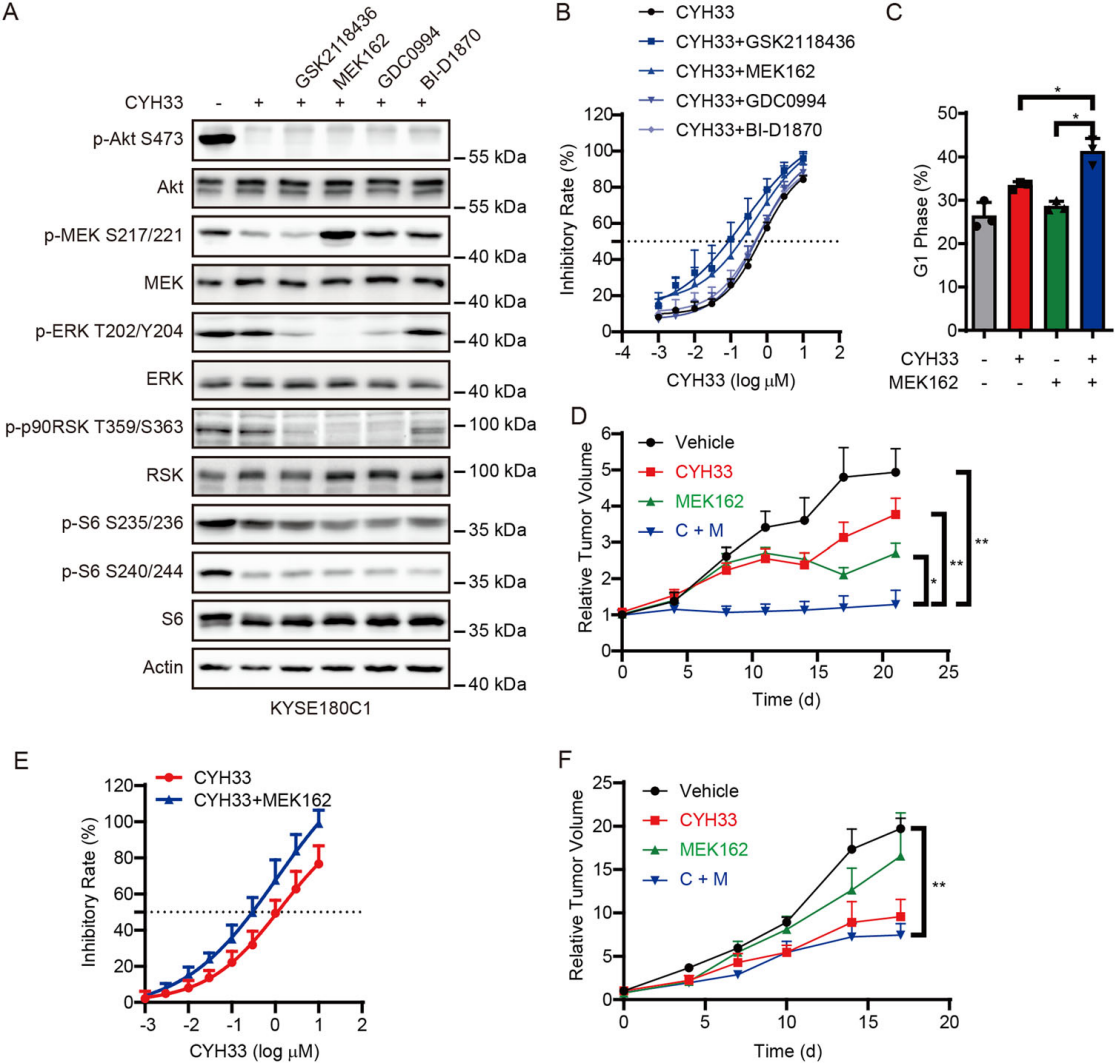
Inhibition of MAPK signaling pathway sensitized KYSE180C cells to CYH33. **A** KYSE180C1 cells were treated with 1 μM CYH33 alone or in combination with 1 μM GSK2118436/MEK162/GDC0994/BI-D1870 for 1 h. Cell lysates were subjected to western blotting with indicated antibodies. **B** KYSE180C1 cells were treated with serially diluted CYH33 alone or concurrently with 1 μM GSK2118436/MEK162/GDC0994/BI-D1870 for 72 h. Cell proliferation was measured with SRB assay. Data were presented as mean + SD (*n* = 3). **C** KYSE180C1 cells were treated with CYH33 (1 μM) and MEK162 (1 μM) alone or concurrently and cell cycle distribution was analyzed by flow cytometry. Percentage of cell population in G1 phase was presented as mean + SD (*n* = 3). Differences between the indicated groups were analyzed using one-way ANOVA with Tukey multiple group comparison test. ^*^P < 0.05. **D** Randomly grouped nude mice bearing KYSE180C1 xenografts were administrated orally with a vehicle control, CYH33 (10 mg/kg), MEK162 (5 mg/kg), or a combination of CYH33 and MEK162 once a day for 21 days (*n* = 6). Tumor volume was measured twice a week. Data were presented as mean + SEM. Differences between the indicated groups were analyzed using one-way ANOVA with Tukey multiple group comparison test. ^**^P < 0.01. **E** KYSE180-H cells were treated with serially diluted CYH33 alone or concurrently with 1 μM MEK162 for 72 h. Cell proliferation was measured with SRB assay. Data were presented as mean + SD (*n* = 3). **F** Randomly grouped nude mice bearing KYSE180-H xenografts were administrated orally with a vehicle control, CYH33 (10 mg/kg), MEK162 (5 mg/kg), or a combination of CYH33 and MEK162 once a day for 17 days (*n* = 3). Tumor volume was measured twice a week. Data were presented as mean + SEM. Differences between the indicated groups were analyzed using one-way ANOVA with Tukey multiple group comparison test. ^*^P < 0.05.

To develop rational drug combinations to circumvent acquired CYH33 resistance, we screened small-molecule inhibitors targeting proteins downstream of HRAS because there is no HRAS-selective inhibitor available. As shown in Fig. 5A, GSK2118436, MEK162, GDC0994, and BI-D1870 at the concentration of 1 μM inhibited their respective target demonstrated as phosphorylated MAPK (MEK), ERK, ribosomal protein S6 kinase A (RSK) or S6. GSK2118436 and MEK162 sensitized KYSE180C1 cells to CYH33 to a greater degree than GDC0994 and BI-D1870, indicating that inhibition of MEK would abrogate *HRAS*^*G12S*^-mediated resistance to CYH33 (Fig. 5B). MEK162 at the concentration of 1 μM also sensitized KYSE180C2 and KYSE180C3 cells to CYH33 (Supplemental Fig. 4A). Consistently, combination of CYH33 and MEK162 enhanced G1 phase arrest in KYSE180C1 cells (Fig. 5C and supplemental Fig. 4B). We next examined the efficacy of CYH33 and MEK162 in mice bearing KYSE180C1 xenografts. CYH33 alone slightly inhibited the growth of KYSE180C1 xenografts, while co-administration of CYH33 and MEK162 potently inhibited tumor growth and resulted in significant reduction in tumor weight compared to the control group (Fig. 5D and Supplemental Fig. 4C). Accordingly, co-administration of CYH33 and MEK162 simultaneously blocked phosphorylation of Akt and ERK, resulting in enhanced inhibition on phosphorylation of S6 (Supplemental Fig. 4D). Similar results were obtained in KYSE180-H cells and xenografts. MEK162 sensitized KYSE180-H cells to CYH33 (Fig. 5E), which was accompanied with reduced phosphorylated ERK (Supplemental Fig. 4E). Co-administration of CYH33 and MEK162 significantly inhibited the growth of KYSE180-H xenografts (Fig. 5F and Supplemental Fig. 4F) with co-current inhibition on phosphorylation of Akt and ERK (Supplemental Fig. 4G). Collectively, inhibition of MEK restored the sensitivity of KYSE180C1 and KYSE180-H cells to CYH33 in vitro and in vivo.

### Combination of CYH33 with MEK/mTORC1/BET inhibitors synergistically suppressed growth of CYH33-resistant cells

**Fig. 6:**
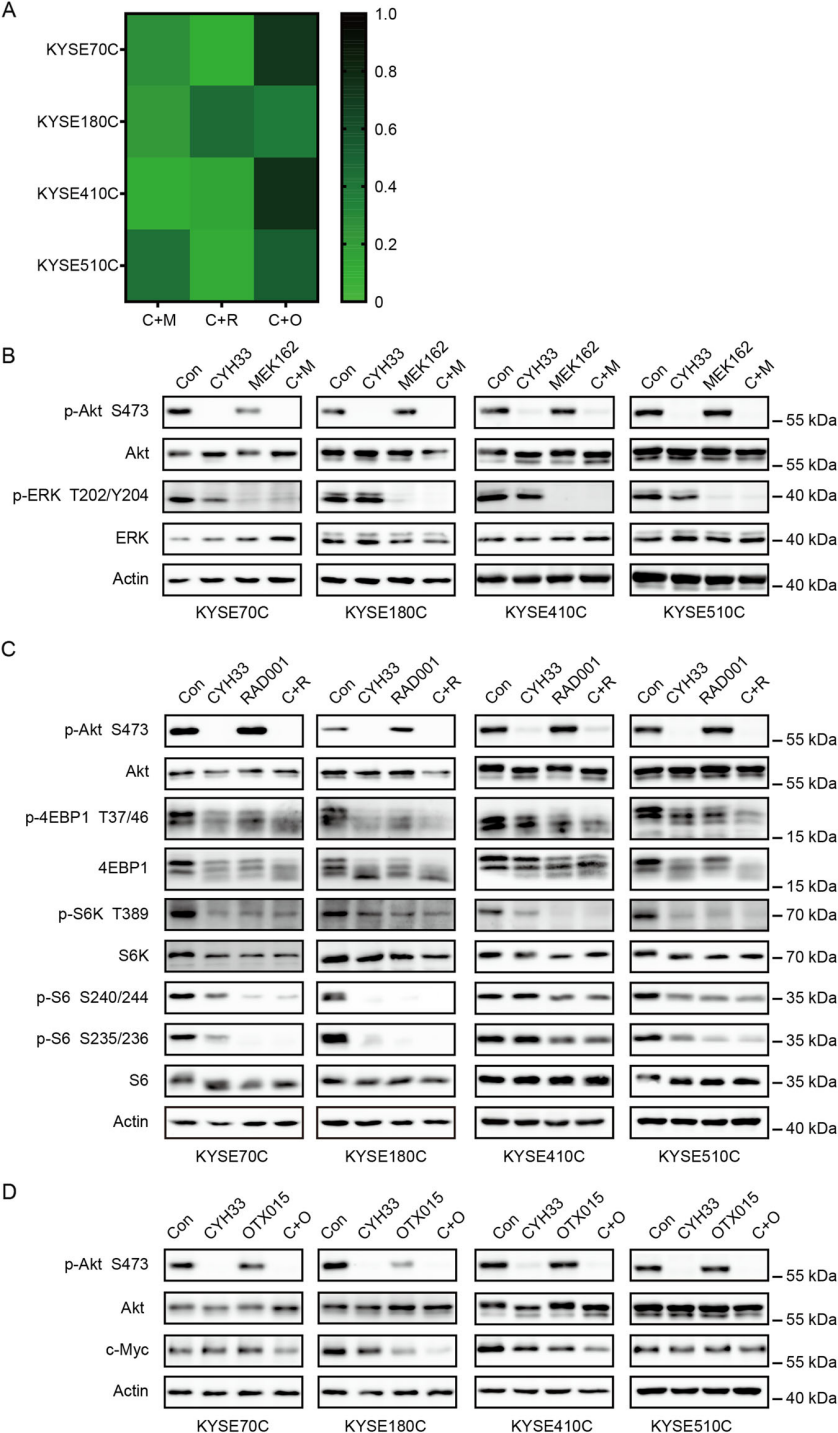
Combination of CYH33 with MEK/mTORC1/BET inhibitors synergistically suppressed growth of CYH33-resistant cells. **A** CYH33-resistant cells were treated with MEK162/RAD001/OTX015 and CYH33 alone or in combination for 72 h. Cell proliferation was measured with SRB assay and combination index (CI) values were calculated by Calcu Syn software (*n* = 3). **B**–**D** CYH33-resistant cells were treated with CYH33 (1 μM, 1 h), MEK162 (1 μM, 1 h) (**B**), RAD001 (1 μM, 1 h) (**C**), or OTX015 (1 μM, 24 h) (**D**) alone or in combination. Cell lysates were then subjected to western blotting with indicated antibodies. Data shown are from at least two independent experiments. C: CYH33, M: MEK162, R: RAD001, O: OTX015.

We found that activation of MAPK pathway, mTORC1, and c-Myc was frequently occurred in CYH33-resistant ESCC cells (Fig. 3). To discover new regimen to overcome acquired resistance to CYH33, combinatorial study of CYH33 with MEK inhibitor (MEK162), mTORC1 inhibitor (RAD001) or bromodomain and extra terminal domain (BET) inhibitor (OTX015) was performed in established resistant cell lines. We examined the anti-proliferative activity of the combinations and calculated the CI using Chou-Talalay method. As expected, we observed synergistic effect with the drug combinations in CYH33-resistant cells, with CI values less than 1 (Fig. 6A and Supplemental Fig. 5A–C). To explore the mechanism underlying the synergism, we dissected signaling pathways targeted by the employed compounds in CYH33-resistant cells. As shown in Fig. 6B, CYH33 and MEK162 at the concentration of 1 μM suppressed phosphorylation of Akt or ERK, respectively. However, combined CYH33 and MEK162 simultaneously blocked activity of both PI3K pathway and MAPK pathway in CYH33-resistant cells. CYH33 partially suppressed phosphorylation of eukaryotic translation initiation facter 4E binding protein 1 (4EBP1), S6K1, and S6, and co-treatment of CYH33 and RAD001 resulted in enhanced inhibition on phosphorylation of 4EBP1 and S6K (Fig. 6C). Overexpression of c-Myc is a common mechanism mediating drug resistance. CYH33 at 1 μM reduced the level of c-Myc protein in KYSE180C and KYSE410C cells. Pretreatment with OTX015 for 23 h enhanced the ability of CYH33 to reduce the level of c-Myc protein in KYSE70C, KYSE180C, and KYSE410C cells (Fig. 6D). Thus, combination of CYH33 with MEK/mTORC1/BET inhibitors synergistically suppressed the proliferation of CYH33-resistant cells.

## Discussion

CYH33 is a novel PI3Kα-selective inhibitor in clinical trials for the advanced solid tumors including ESCC based on its promising efficacy in preclinical settings (Fig. 1A)^28,44^. However, acquired resistance induced by continuous PI3Kα inhibition would inevitably occur and compromise the therapeutic efficacy. In this study, we found gained mutations and amplification of chromosome segments in established CYH33-resistant ESCC cells, which is associated with PI3Kα-independent activation of mTORC1, MAPK, and c-Myc signaling. Especially, gain-of-function *HRAS*^*G12S*^ mutant was able to confer resistance to CYH33 in ESCC cells, which could be abrogated by co-current inhibition of MAPK and PI3Kα. Similarly, combination of RAD001 and OTX015 with CYH33 displayed synergistic activity against the proliferation of the resistant cells.

The potential mechanisms to mediate acquired resistance upon inhibition of PI3Kα have been studied in different types of cancers, while the global genomic sequencing has not been reported in ESCC cells resistant to PI3Kα inhibitors. By sequencing the whole genome of the resistant and the parental cells, we revealed frequent amplified or mutated genes gained in resistant cells, which are involved in regulation of cell survival and proliferation. Accordingly, we found that mTORC1, MAPK, and c-Myc pathways were hyper-activated in resistant cells in a PI3Kα-independent manner. Mutations in the target protein have been frequently found to induce resistance. For example, *EGFR*^*T790M*^ mutant mediates resistance to the first- and second-generation of EGFR inhibitors^45^, while C797S mutant mediates resistance to inhibitors targeting T790M mutant^46^. However, mutation in *PIK3CA* was not identified in resistant cells, which is consistent with inhibition of Akt phosphorylation in resistant cells by PI3Kα inhibitors. Of particular interest, we identified *HRAS*^*G12S*^ mutant in resistant KYSE180C cells after continuous exposure to CYH33. This mutation was induced by inhibition of PI3Kα but not pre-existed in sub-clone of KYSE180 cells, because no mutation was found by deep sequencing of the *HRAS* gene in parental cells (data not shown). We found that *HRAS*^*G12S*^ mutant was associated with resistance to PI3Kα inhibitors in ESCC cells by the three lines of evidences: firstly, ectopic expression of HRAS^G12S^ in ESCC cells significantly enhanced MAPK signaling and rendered resistance to CYH33; secondly, knockdown of HRAS^G12S^ or inhibition of signaling downstream of HRAS sensitized resistant cells to CYH33; thirdly, combination of CYH33 and MEK inhibitor synergistically inhibited the growth of xenografts that have originated from resistant cells harboring *HRAS*^*G12S*^. Though *HRAS*^*G12S*^ mutation was rarely found in ESCC, 0.6–6% of ESCC harbors gain-of-function mutation in *KRAS*^2,47^. Our study also suggested that concurrent inhibition of PI3Kα and RAS/MEK is worthwhile to be considered in the treatment of ESCC harboring mutation in *KRAS* or *HRAS*.

In consistency with PI3Kα-independent activation of mTORC1, MAPK, and c-Myc signaling in CYH33-resistant ESCC cells, simultaneous inhibition of PI3Kα and mTORC1, MEK, or BET displayed synergistic activity against the proliferation of the resistant cells. These results also confirmed that activation of mTORC1, MAPK or c-Myc signaling might contribute to resistance to CYH33. Actually, activation of mTORC1, MAPK, or c-Myc signaling has been reported to mediate adaptive resistance to PI3K inhibition in cancers that have originated from different tissue types. Persistent mTORC1 activation was found to compromise alpelisib efficacy in breast cancer, while inhibition of mTORC1 with RAD001 enhanced the efficacy of PI3Kα inhibitors and delayed onset of resistance^43^. Akt-independent activation of mTORC1 was probably mediated by c-Jun/c-Fos-induced overexpression of AXL receptor tyrosine kinase (AXL)^48^ and the following AXL- phospholipase C gamma (PLCγ)–protein kinase C (PKC) axis^20^. Acquired resistance to GDC-0941 in SUM-159 breast cancer cells was found to be associated with a focal amplification in the *MYC* locus and inhibition of BET with JQ1 sensitized resistant cells to GDC-0941 treatment^49^. We also previously reported that decrease in phosphorylated ERK indicated the therapeutic efficacy of CYH33 in breast cancer^26^. These studies suggest that continuous exposure to PI3Kα inhibitor frequently induces activation of mTORC1, MAPK, or c-Myc signaling, which may contribute to resistance to PI3K inhibitors. Thus, Co-current targeting one of these pathways and PI3K would circumvent the acquired resistance. Multiple oncogenic pathways, such as mTORC1 signaling, E2F targets, KRAS signaling, and the estrogen response have been found up-regulated in breast cancer cells with acquired resistance to CYH33^29^. Targeting transmembrane transporter exportin 1 (XPO1) might overcome the resistance to CYH33 in breast cancer cells, which was associated with accumulation of p53 in the nucleus^29^. However, *TP53* mutation was frequently detected in ESCC. Different strategy might be required to overcome adaptive resistance to CYH33 in different cancer types.

In summary, genomic and transcriptomic profiling revealed that activation of mTORC1, MAPK, and c-Myc signaling was associated with adaptive resistance to CYH33 in ESCC cells, and combination of CYH33 and RAD001, MEK162, or OTX015 demonstrated synergistic activity against these resistant cells. Our findings provide a mechanistic rationale to test PI3Kα inhibitors in combination with drugs targeting mTORC1, MEK, or BET in treating ESCC relapsed from monotherapy.

## Supplementary information

Supplementary Figure Legends.

Supplementary Figures.

Table S1

Table S2

Table S3

Table S4

Table S5

Table S6

Table S7
