# Supplementary material for: Adaptive resistance to PI3Kα-selective inhibitor CYH33 is mediated by genomic and transcriptomic alterations in ESCC cells
Source: Cell Death Dis. 2021 Jan 14;12(1):85. doi: 10.1038/s41419-020-03370-4 (PMC7809409; doi:10.1038/s41419-020-03370-4)
Supplement: Supplementary file 1 — Supplementary Figure Legends. [file 41419_2020_3370_MOESM1_ESM.docx]

**Supplementary Figures Legends**

**Figure S1. ESCC cells developed acquired resistance after continuous exposure to PI3Kα inhibition.**

(A) Parental and resistant cells were incubated with alpelisib for 72 h and cell proliferation was measured with SRB assay. Resistance factor (RF) was calculated by the ratio of GI_50_ obtained in CYH33-resistant cells and that in parental cells (n=3). (B) Parental and resistant cells were treated with alpelisib at the indicated concentrations for 72 h and cell cycle distribution was analyzed by flow cytometry (n=3). (C) Parental and resistant cells were treated with CYH33 for 72 h and cell cycle distribution was analyzed by flow cytometry. Representative pictures of cell cycle were presented. Data were presented as mean + SD. Differences between indicated groups were analyzed using one-way ANOVA with Tukey multiple group comparison test. * *P* < 0.05, ** *P* < 0.01.

**Figure S2. Dysregulated pathways in CYH33-resistant ESCC cells.**

Global gene expression was profiled with RNA-Seq in CYH33-resistant cells as well as their parental cells. Differentially expressed genes were collected for the KEGG pathway analysis. Top enriched pathways were shown in the bubble diagram.

**Figure S3. Gain of function mutation in *HRAS* rendered resistance to PI3Kα inhibitors in ESCC cells.**

(A) KYSE180-V and KYSE180-H cells were treated with alpelisib for 72 h and cell proliferation was measured with SRB assay (n=3). (B) KYSE180-V and KYSE180-H cells were incubated with CYH33 for 24 h and cell cycle distribution was analyzed by flow cytometry. Representative pictures of cell cycle were presented. (C) KYSE180 cells were transfected with plasmid expressing wild type HRAS (180-WT), HRAS^G12S^ mutant (180-M) or a vehicle plasmid (180-V), and cell lysates were subjected to Western blotting with indicated antibodies. 180-V, 180-WT and 180-M cells were treated with CYH33 for 72 h and cell proliferation was measured with SRB assay (n=3). (D) KYSE70, KYSE410 and KYSE510 cells, transfected with plasmid expressing HRAS^G12S^ mutant (KYSE70-H, KYSE410-H and KYSE510-H) or a vehicle plasmid (KYSE70-V, KYSE410-V and KYSE510-V), were treated with CYH33 for 72 h and cell proliferation was measured with SRB assay (n=3). Data were presented as mean + SD.

**Figure S4. Inhibition of MAPK signaling pathway sensitized KYSE180C cells to CYH33**

(A) KSYE180C2 and KYSE180C3 cells were treated with serially diluted CYH33 alone or concurrently with 1 μM MEK162 for 72 h. Cell proliferation was measured with SRB assay. Data were presented as mean + SD (n=3). (B) KYSE180C1 cells were treated with CYH33 (1 μM) and MEK162 (1 μM) alone or concurrently and cell cycle distribution was analyzed by flow cytometry. Representative pictures of cell cycle were presented. (C) Randomly grouped nude mice bearing KYSE180C1 xenografts were administrated orally with a vehicle control, CYH33 (10 mg/kg), MEK162 (5 mg/kg), or a combination of CYH33 and MEK162 once a day for 21 d. Body weight was measured twice a week and tumor weight was measured at the end of the experiment. Data were presented as mean + SEM (n=6). Differences between indicated groups were analyzed using one-way ANOVA with Tukey multiple group comparison test. ** *P* < 0.01. (D) Immunoblot analysis of KYSE180C1 xenografts harvested 2 h after the last drug treatment. (E) KYSE180-H cells were treated with MEK162 at indicated concentrations for 1 h, and cell lysates were subjected to Western blotting with indicated antibodies. (F) Randomly grouped nude mice bearing KYSE180-H xenografts were administrated orally with a vehicle control, CYH33 (10 mg/kg), MEK162 (5 mg/kg), or a combination of CYH33 and MEK162 once a day for 17 d. Body weight was measured twice a week and tumor weight was measured at the end of the experiment. Data were presented as mean + SEM (n=3). Differences between indicated groups were analyzed using one-way ANOVA with Tukey multiple group comparison test. * *P* < 0.05. (G) Immunoblot analysis of KYSE180-H xenografts harvested 2 h after the last drug treatment.

**Figure S5. Combination of CYH33 with MEK/mTOR/BET inhibitors synergistically suppressed growth of CYH33-resistant cells.**

(A) CYH33-resistant cells were treated with MEK162 and CYH33 alone or in combination for 72 hours. Cell proliferation was measured with SRB assay (n=3). (B) CYH33-resistant cells were treated with RAD001 and CYH33 alone or in combination for 72 hours. Cell proliferation was measured with SRB assay (n=3). (C) CYH33-resistant cells were treated with OTX015 and CYH33 alone or in combination for 72 hours. Cell proliferation was measured with SRB assay (n=3). Data were presented as mean + SD.

**Supplementary Tables**

**Table S1-S4. High-confidence non-synonymous somatic mutations in KYSE70C, KYSE180C, KYSE410C and KYSE510C cells.**

**Table S5-S7. Amplification of chromosome segments in KYSE180C, KYSE410C and KYSE510C cells.**
