# Supplementary figures and images for: Adaptive resistance to PI3Kα-selective inhibitor CYH33 is mediated by genomic and transcriptomic alterations in ESCC cells
Source: Cell Death Dis. 2021 Jan 14;12(1):85. doi: 10.1038/s41419-020-03370-4 (PMC7809409; doi:10.1038/s41419-020-03370-4)

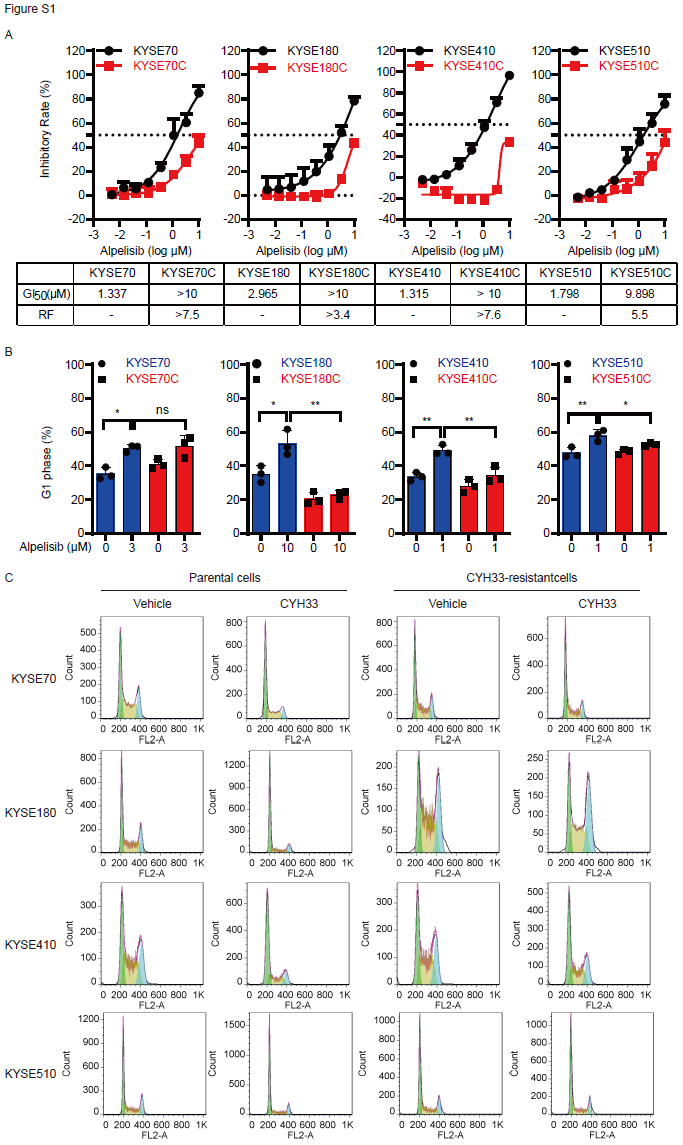


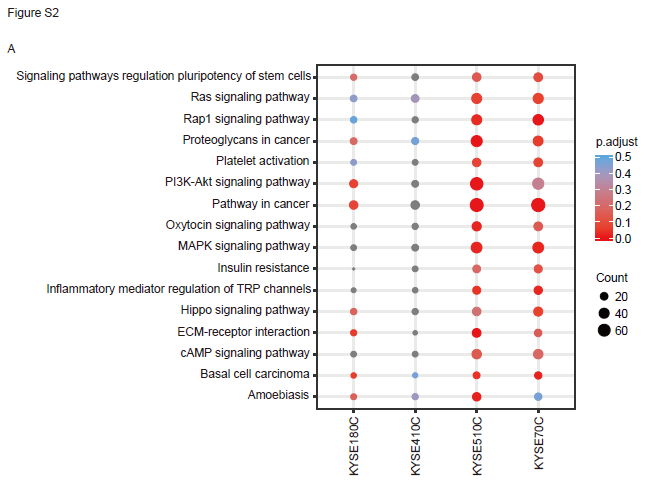


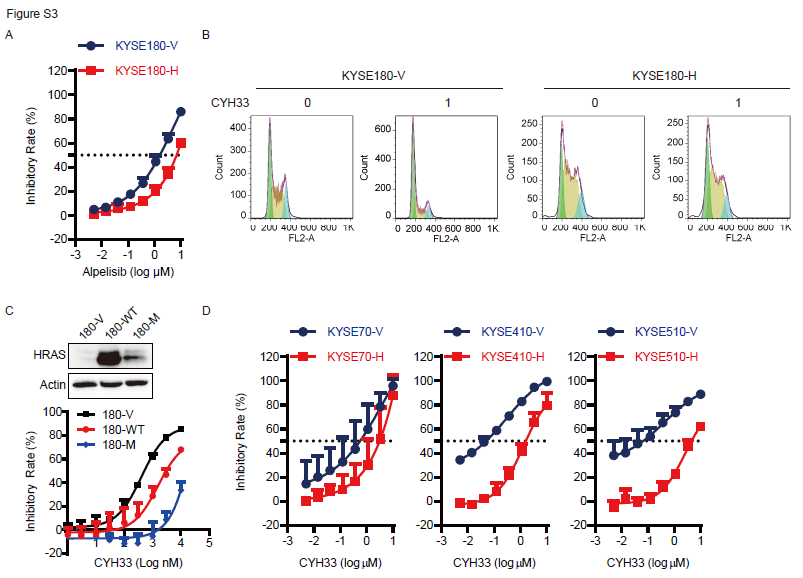


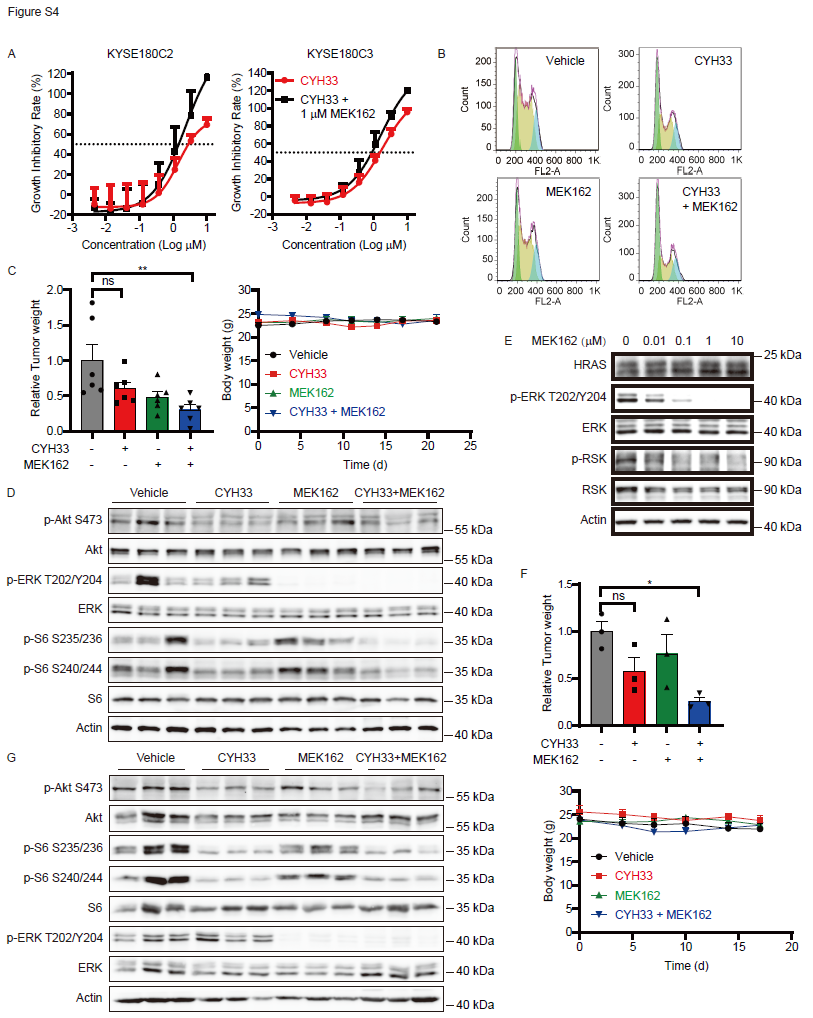


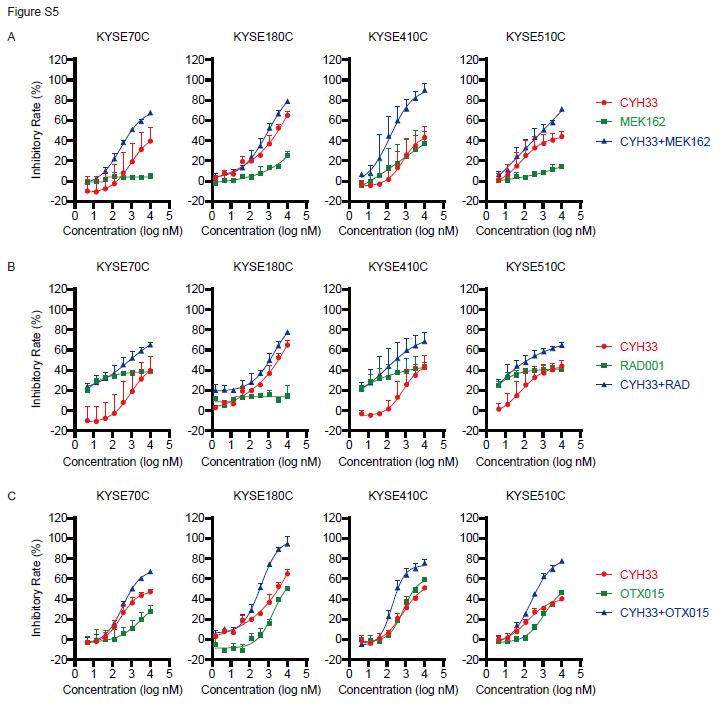

Supplement: Supplementary file 2 — Supplementary Figures. [file 41419_2020_3370_MOESM2_ESM.docx]
