# Supplementary material for: Adaptive resistance to PI3Kα-selective inhibitor CYH33 is mediated by genomic and transcriptomic alterations in ESCC cells
Source: Cell Death Dis. 2021 Jan 14;12(1):85. doi: 10.1038/s41419-020-03370-4 (PMC7809409; doi:10.1038/s41419-020-03370-4)
Supplement: Supplementary file 3 — Table S1 [file 41419_2020_3370_MOESM3_ESM.docx]

| #CHROM | POS | REF | ALT | Gene | ExonicFunc.refGene |
| --- | --- | --- | --- | --- | --- |
| chr10 | 27499896 | TTCCAG  GAGGTA  CTTGA | T | ACBD5 | frameshift deletion |
| chr16 | 68591905 | CAG | C | ZFP90 | frameshift deletion |
| chr9 | 87339233 | TAGG | T | NTRK2 | nonframeshift deletion |
| chr3 | 195513398 | C | CGGTGA  CAGGAA  GAGGGG  TGGTGT  GACCTG  TGGATG  CTGAGG  AAGTGC  T | MUC4 | nonframeshift insertion |
| chr1 | 19951956 | C | A | MINOS1 | nonsynonymous SNV |
| chr1 | 113653003 | C | G | LRIG2 | nonsynonymous SNV |
| chr1 | 146687339 | C | A | FMO5 | nonsynonymous SNV |
| chr1 | 207647202 | G | A | CR2 | nonsynonymous SNV |
| chr1 | 207649704 | G | C | CR2 | nonsynonymous SNV |
| chr1 | 244528037 | G | A | C1orf100 | nonsynonymous SNV |
| chr2 | 73677678 | G | A | ALMS1 | nonsynonymous SNV |
| chr2 | 73677679 | T | G | ALMS1 | nonsynonymous SNV |
| chr2 | 216256459 | G | T | FN1 | nonsynonymous SNV |
| chr3 | 45160071 | A | C | CDCP1 | nonsynonymous SNV |
| chr6 | 51612912 | C | G | PKHD1 | nonsynonymous SNV |
| chr7 | 1037317 | G | A | C7orf50 | nonsynonymous SNV |
| chr7 | 5645020 | G | A | FSCN1 | nonsynonymous SNV |
| chr7 | 20198020 | G | C | MACC1 | nonsynonymous SNV |
| chr7 | 70800586 | G | T | WBSCR17 | nonsynonymous SNV |
| chr9 | 6421131 | G | A | UHRF2 | nonsynonymous SNV |
| chr9 | 8501000 | C | G | PTPRD | nonsynonymous SNV |
| chr9 | 90535442 | G | A | SPATA31C1 | nonsynonymous SNV |
| chr10 | 23481502 | G | A | PTF1A | nonsynonymous SNV |
| chr10 | 135440123 | C | T | FRG2B | nonsynonymous SNV |
| chr11 | 6637237 | T | A | TPP1 | nonsynonymous SNV |
| chr11 | 44077628 | C | G | ACCSL | nonsynonymous SNV |
| chr11 | 58919431 | C | A | FAM111A | nonsynonymous SNV |
| chr11 | 61017203 | G | T | PGA5 | nonsynonymous SNV |
| chr11 | 106558362 | C | G | GUCY1A2 | nonsynonymous SNV |
| chr12 | 20787945 | A | T | PDE3A | nonsynonymous SNV |
| chr12 | 50152526 | G | C | TMBIM6 | nonsynonymous SNV |
| chr12 | 68710020 | C | T | MDM1 | nonsynonymous SNV |
| chr14 | 60005449 | C | G | CCDC175 | nonsynonymous SNV |
| chr17 | 11593737 | G | T | DNAH9 | nonsynonymous SNV |
| chr17 | 12847451 | G | A | ARHGAP44 | nonsynonymous SNV |
| chr17 | 17057665 | C | T | MPRIP | nonsynonymous SNV |
| chr18 | 3814144 | G | A | DLGAP1 | nonsynonymous SNV |
| chr18 | 14106348 | T | C | ZNF519 | nonsynonymous SNV |
| chr19 | 1792042 | C | A | ATP8B3 | nonsynonymous SNV |
| chr19 | 11618538 | G | A | ECSIT | nonsynonymous SNV |
| chr19 | 57666644 | C | T | DUXA | nonsynonymous SNV |
| chr20 | 42744331 | C | A | JPH2 | nonsynonymous SNV |
| chr20 | 55948543 | G | T | RAE1 | nonsynonymous SNV |
| chr22 | 16277757 | C | T | POTEH | nonsynonymous SNV |
| chr22 | 23915736 | G | A | IGLL1 | nonsynonymous SNV |
| chr22 | 39381841 | G | A | APOBEC3B | nonsynonymous SNV |
| chrX | 104464220 | G | A | TEX13A | nonsynonymous SNV |
| chr9 | 120053634 | G | A | ASTN2 | stopgain |
| chr16 | 67184112 | G | A | B3GNT9 | stopgain |
| chr18 | 67614202 | C | T | CD226 | stopgain |
| chr19 | 20229554 | C | G | ZNF90 | stopgain |
