# Supplementary material for: Adaptive resistance to PI3Kα-selective inhibitor CYH33 is mediated by genomic and transcriptomic alterations in ESCC cells
Source: Cell Death Dis. 2021 Jan 14;12(1):85. doi: 10.1038/s41419-020-03370-4 (PMC7809409; doi:10.1038/s41419-020-03370-4)
Supplement: Supplementary file 4 — Table S2 [file 41419_2020_3370_MOESM4_ESM.docx]

| #CHROM | POS | REF | ALT | Gene.refGene | ExonicFunc.refGene |
| --- | --- | --- | --- | --- | --- |
| chr6 | 43608158 | GC | G | MAD2L1BP | frameshift deletion |
| chr1 | 34238207 | C | G | CSMD2 | nonsynonymous SNV |
| chr1 | 153945876 | G | A | CREB3L4 | nonsynonymous SNV |
| chr1 | 237811768 | T | A | RYR2 | nonsynonymous SNV |
| chr2 | 9515029 | G | A | ASAP2 | nonsynonymous SNV |
| chr2 | 73676692 | A | G | ALMS1 | nonsynonymous SNV |
| chr2 | 133541121 | C | A | NCKAP5 | nonsynonymous SNV |
| chr2 | 218683314 | G | C | TNS1 | nonsynonymous SNV |
| chr4 | 62935844 | G | T | ADGRL3 | nonsynonymous SNV |
| chr4 | 174292476 | G | C | SAP30 | nonsynonymous SNV |
| chr5 | 140745675 | C | T | PCDHGA5 | nonsynonymous SNV |
| chr6 | 34738088 | A | T | SNRPC | nonsynonymous SNV |
| chr7 | 64388564 | A | T | ZNF273 | nonsynonymous SNV |
| chr7 | 114284770 | C | A | FOXP2 | nonsynonymous SNV |
| chr8 | 144358478 | C | A | GLI4 | nonsynonymous SNV |
| chr9 | 32632118 | C | G | TAF1L | nonsynonymous SNV |
| chr9 | 40703187 | C | T | SPATA31A3 | nonsynonymous SNV |
| chr10 | 49450352 | A | G | FRMPD2 | nonsynonymous SNV |
| chr11 | 534289 | C | T | HRAS | nonsynonymous SNV |
| chr11 | 95578236 | G | A | MTMR2 | nonsynonymous SNV |
| chr12 | 113837490 | G | C | SDS | nonsynonymous SNV |
| chr13 | 110436659 | G | T | IRS2 | nonsynonymous SNV |
| chr17 | 4721622 | C | T | PLD2 | nonsynonymous SNV |
| chr17 | 19247017 | C | A | B9D1 | nonsynonymous SNV |
| chr18 | 13116437 | A | C | CEP192 | nonsynonymous SNV |
| chr19 | 21990580 | C | G | ZNF43 | nonsynonymous SNV |
| chr5 | 115813729 | G | A | SEMA6A | stopgain |
| chr9 | 86368260 | T | TGCATCATAA  CCTATTTCAA  TACATTCTTT  ATATTGATCC  TTGTATTTAG  CTTGTTGTTT  TTCTGGTCAA  ACTTTTGCTA  TATCTAATCT  AAGAGTTATT  AATTTAGTTT  TAACTACAAT  ATCTAT  ATG | GKAP1 | stopgain |
| chr9 | 115759735 | C | A | ZNF883 | stopgain |
