# Supplementary material for: Adaptive resistance to PI3Kα-selective inhibitor CYH33 is mediated by genomic and transcriptomic alterations in ESCC cells
Source: Cell Death Dis. 2021 Jan 14;12(1):85. doi: 10.1038/s41419-020-03370-4 (PMC7809409; doi:10.1038/s41419-020-03370-4)
Supplement: Supplementary file 5 — Table S3 [file 41419_2020_3370_MOESM5_ESM.docx]

| #CHROM | POS | REF | ALT | Gene.refGene | ExonicFunc.refGene |
| --- | --- | --- | --- | --- | --- |
| chr1 | 1688095 | TC | T | NADK | frameshift deletion |
| chr8 | 139180207 | CCAGG | C | FAM135B | frameshift deletion |
| chr22 | 24622164 | T | TC | GGT5 | frameshift insertion |
| chr19 | 41173898 | TTGCTGTTGC | T | NUMBL | nonframeshift deletion |
| chr19 | 55645525 | C | CGGAGCT  GTTCCTC  CCCCA | TNNT1;TNNT1 | nonframeshift insertion |
| chr1 | 21807427 | G | T | NBPF3 | nonsynonymous SNV |
| chr1 | 67633804 | A | G | IL23R | nonsynonymous SNV |
| chr1 | 165712453 | T | C | TMCO1 | nonsynonymous SNV |
| chr1 | 243828126 | G | T | AKT3 | nonsynonymous SNV |
| chr2 | 74742967 | C | G | TLX2 | nonsynonymous SNV |
| chr2 | 98458331 | T | A | TMEM131 | nonsynonymous SNV |
| chr2 | 220480806 | G | T | STK11IP | nonsynonymous SNV |
| chr5 | 35065307 | G | T | PRLR | nonsynonymous SNV |
| chr5 | 140594363 | G | C | PCDHB13 | nonsynonymous SNV |
| chr5 | 140605248 | G | T | PCDHB14 | nonsynonymous SNV |
| chr6 | 64431473 | A | T | EYS | nonsynonymous SNV |
| chr6 | 117894713 | G | A | GOPC | nonsynonymous SNV |
| chr7 | 82581525 | G | C | PCLO | nonsynonymous SNV |
| chr7 | 98786061 | G | T | KPNA7 | nonsynonymous SNV |
| chr7 | 141750557 | A | C | MGAM | nonsynonymous SNV |
| chr8 | 143436046 | T | C | TSNARE1 | nonsynonymous SNV |
| chr9 | 25677767 | T | G | TUSC1 | nonsynonymous SNV |
| chr9 | 91686132 | G | T | SHC3 | nonsynonymous SNV |
| chr10 | 106125688 | A | T | CFAP58 | nonsynonymous SNV |
| chr11 | 30032905 | C | T | KCNA4 | nonsynonymous SNV |
| chr11 | 92565010 | T | C | FAT3 | nonsynonymous SNV |
| chr11 | 133795794 | C | A | IGSF9B | nonsynonymous SNV |
| chr12 | 11244527 | G | C | TAS2R43 | nonsynonymous SNV |
| chr12 | 54063092 | T | A | ATP5G2 | nonsynonymous SNV |
| chr12 | 124816913 | C | T | NCOR2 | nonsynonymous SNV |
| chr13 | 20797346 | C | T | GJB6 | nonsynonymous SNV |
| chr16 | 20838486 | C | T | LOC81691 | nonsynonymous SNV |
| chr17 | 10432240 | T | G | MYH2 | nonsynonymous SNV |
| chr17 | 27239774 | C | A | PHF12 | nonsynonymous SNV |
| chr18 | 29487497 | C | G | TRAPPC8 | nonsynonymous SNV |
| chr19 | 13346050 | G | T | CACNA1A | nonsynonymous SNV |
| chr19 | 15905019 | G | A | OR10H5 | nonsynonymous SNV |
| chr19 | 49937116 | C | T | SLC17A7 | nonsynonymous SNV |
| chr19 | 51021476 | C | A | LRRC4B | nonsynonymous SNV |
| chr19 | 52220232 | C | T | HAS1 | nonsynonymous SNV |
| chr20 | 45867689 | T | A | ZMYND8 | nonsynonymous SNV |
| chr20 | 51872809 | C | A | TSHZ2 | nonsynonymous SNV |
| chr20 | 56071388 | A | G | CTCFL | nonsynonymous SNV |
| chr20 | 60775876 | G | C | MTG2 | nonsynonymous SNV |
| chr22 | 24579157 | G | T | SUSD2 | nonsynonymous SNV |
| chr22 | 44586519 | C | A | PARVG | nonsynonymous SNV |
| chrX | 50376671 | G | A | SHROOM4 | nonsynonymous SNV |
| chrX | 112058717 | G | T | AMOT | nonsynonymous SNV |
| chr6 | 55739504 | C | A | BMP5 | stopgain |
| chr6 | 161056330 | CA | C | LPA | stopgain |
| chr11 | 55579389 | C | A | OR5L1 | stopgain |
| chr17 | 74718008 | C | T | JMJD6 | stopgain |
