# Supplementary material for: Adaptive resistance to PI3Kα-selective inhibitor CYH33 is mediated by genomic and transcriptomic alterations in ESCC cells
Source: Cell Death Dis. 2021 Jan 14;12(1):85. doi: 10.1038/s41419-020-03370-4 (PMC7809409; doi:10.1038/s41419-020-03370-4)
Supplement: Supplementary file 6 — Table S4 [file 41419_2020_3370_MOESM6_ESM.docx]

| #CHROM | POS | REF | ALT | Gene.refGene | ExonicFunc.refGene |
| --- | --- | --- | --- | --- | --- |
| chr3 | 136664504 | AC | A | NCK1 | frameshift deletion |
| chr6 | 64431581 | AG | A | EYS | frameshift deletion |
| chr6 | 132031081 | AAG | A | CTAGE9 | frameshift deletion |
| chr7 | 100551481 | AT | A | MUC3A | frameshift deletion |
| chr9 | 107361450 | AGC | A | OR13C5 | frameshift deletion |
| chr3 | 153993965 | TGTAATATCCATCCTGGAATCGTGGCGGAAA | T | DHX36 | nonframeshift deletion |
| chr16 | 67229793 | ACAGCAGCAGCAGCAGCAGCAGCAGCAG | A | E2F4 | nonframeshift deletion |
| chr1 | 6529182 | T | TTCC | PLEKHG5 | nonframeshift insertion |
| chr19 | 46299138 | T | TCTCCTCGCCCTCCTC | RSPH6A | nonframeshift insertion |
| chr22 | 50716590 | T | TGTGAGAGGGCCACATGTATGGGGA | PLXNB2 | nonframeshift insertion |
| chr1 | 17405849 | G | A | PADI2 | nonsynonymous SNV |
| chr1 | 22055204 | G | C | USP48 | nonsynonymous SNV |
| chr1 | 22056316 | G | C | USP48 | nonsynonymous SNV |
| chr1 | 23219384 | G | T | EPHB2 | nonsynonymous SNV |
| chr1 | 24078196 | C | A | TCEB3 | nonsynonymous SNV |
| chr1 | 24288077 | G | C | PNRC2 | nonsynonymous SNV |
| chr1 | 32623896 | C | G | KPNA6 | nonsynonymous SNV |
| chr1 | 36219430 | C | A | CLSPN | nonsynonymous SNV |
| chr1 | 36933436 | C | T | CSF3R | nonsynonymous SNV |
| chr1 | 45274520 | G | A | BTBD19 | nonsynonymous SNV |
| chr1 | 98386472 | G | T | DPYD | nonsynonymous SNV |
| chr1 | 101200183 | G | A | VCAM1 | nonsynonymous SNV |
| chr1 | 120057135 | C | G | HSD3B1 | nonsynonymous SNV |
| chr1 | 152636873 | G | A | LCE2D | nonsynonymous SNV |
| chr1 | 158815726 | C | T | MNDA | nonsynonymous SNV |
| chr1 | 171077322 | G | A | FMO3 | nonsynonymous SNV |
| chr1 | 182026583 | G | A | ZNF648 | nonsynonymous SNV |
| chr1 | 183097764 | G | C | LAMC1 | nonsynonymous SNV |
| chr1 | 202128595 | G | A | PTPN7 | nonsynonymous SNV |
| chr1 | 202130639 | C | A | PTPN7 | nonsynonymous SNV |
| chr1 | 202302625 | C | G | UBE2T | nonsynonymous SNV |
| chr1 | 222791550 | C | G | MIA3 | nonsynonymous SNV |
| chr1 | 241797401 | C | G | CHML | nonsynonymous SNV |
| chr2 | 10074108 | C | T | TAF1B | nonsynonymous SNV |
| chr2 | 21365413 | A | T | TDRD15 | nonsynonymous SNV |
| chr2 | 27708280 | C | G | IFT172 | nonsynonymous SNV |
| chr2 | 29358508 | G | T | CLIP4 | nonsynonymous SNV |
| chr2 | 70315482 | G | A | PCBP1 | nonsynonymous SNV |
| chr2 | 72360424 | C | G | CYP26B1 | nonsynonymous SNV |
| chr2 | 95753226 | G | A | MRPS5 | nonsynonymous SNV |
| chr2 | 143715237 | C | T | KYNU | nonsynonymous SNV |
| chr2 | 160993977 | G | C | ITGB6 | nonsynonymous SNV |
| chr2 | 170366891 | G | A | KLHL41 | nonsynonymous SNV |
| chr2 | 170753020 | G | T | UBR3 | nonsynonymous SNV |
| chr2 | 178081419 | C | T | HNRNPA3 | nonsynonymous SNV |
| chr2 | 179418820 | C | T | TTN | nonsynonymous SNV |
| chr2 | 179424422 | C | T | TTN | nonsynonymous SNV |
| chr2 | 196771717 | C | G | DNAH7 | nonsynonymous SNV |
| chr2 | 202131315 | G | A | CASP8 | nonsynonymous SNV |
| chr3 | 108149754 | C | G | MYH15 | nonsynonymous SNV |
| chr3 | 110791297 | C | T | PVRL3 | nonsynonymous SNV |
| chr3 | 124538627 | G | A | ITGB5 | nonsynonymous SNV |
| chr4 | 3021518 | T | G | GRK4 | nonsynonymous SNV |
| chr4 | 7056386 | G | C | TADA2B | nonsynonymous SNV |
| chr4 | 17528649 | G | C | CLRN2 | nonsynonymous SNV |
| chr5 | 137206435 | C | T | MYOT | nonsynonymous SNV |
| chr5 | 139876752 | C | G | ANKHD1,ANKHD1-EIF4EBP3 | nonsynonymous SNV |
| chr5 | 140574103 | T | G | PCDHB10 | nonsynonymous SNV |
| chr5 | 140789642 | G | A | PCDHGB6 | nonsynonymous SNV |
| chr5 | 150275635 | G | C | ZNF300 | nonsynonymous SNV |
| chr6 | 27805947 | C | A | HIST1H2AK | nonsynonymous SNV |
| chr6 | 36262069 | G | T | PNPLA1 | nonsynonymous SNV |
| chr6 | 40360187 | G | A | LRFN2 | nonsynonymous SNV |
| chr6 | 125621799 | G | A | HDDC2 | nonsynonymous SNV |
| chr6 | 150111117 | G | C | PCMT1 | nonsynonymous SNV |
| chr6 | 159655452 | T | C | FNDC1 | nonsynonymous SNV |
| chr6 | 169634891 | C | T | THBS2 | nonsynonymous SNV |
| chr7 | 4798801 | C | T | FOXK1 | nonsynonymous SNV |
| chr7 | 44153789 | G | A | AEBP1 | nonsynonymous SNV |
| chr7 | 72400549 | C | G | POM121 | nonsynonymous SNV |
| chr7 | 150554237 | G | T | AOC1 | nonsynonymous SNV |
| chr8 | 8750331 | C | G | MFHAS1 | nonsynonymous SNV |
| chr8 | 9413460 | C | G | TNKS | nonsynonymous SNV |
| chr8 | 10480543 | C | T | RP1L1 | nonsynonymous SNV |
| chr8 | 57129969 | G | C | CHCHD7 | nonsynonymous SNV |
| chr8 | 139668150 | A | T | COL22A1 | nonsynonymous SNV |
| chr8 | 145724434 | C | T | PPP1R16A | nonsynonymous SNV |
| chr9 | 2804335 | G | A | PUM3 | nonsynonymous SNV |
| chr9 | 139302353 | G | C | SDCCAG3 | nonsynonymous SNV |
| chr10 | 51585180 | C | G | NCOA4 | nonsynonymous SNV |
| chr10 | 72014886 | C | T | NPFFR1 | nonsynonymous SNV |
| chr10 | 96454786 | G | A | CYP2C18 | nonsynonymous SNV |
| chr10 | 104638624 | C | G | AS3MT | nonsynonymous SNV |
| chr10 | 134621949 | C | A | CFAP46 | nonsynonymous SNV |
| chr11 | 6452541 | C | T | HPX | nonsynonymous SNV |
| chr11 | 55587929 | C | A | OR5D18 | nonsynonymous SNV |
| chr11 | 59271381 | C | A | OR4D11 | nonsynonymous SNV |
| chr11 | 62287855 | C | A | AHNAK | nonsynonymous SNV |
| chr11 | 62327234 | T | C | EEF1G | nonsynonymous SNV |
| chr11 | 62459942 | C | T | BSCL2 | nonsynonymous SNV |
| chr11 | 75590964 | C | G | UVRAG | nonsynonymous SNV |
| chr11 | 77813965 | C | G | ALG8 | nonsynonymous SNV |
| chr11 | 101937338 | G | C | C11orf70 | nonsynonymous SNV |
| chr11 | 119549304 | G | A | PVRL1 | nonsynonymous SNV |
| chr12 | 46756147 | T | A | SLC38A2 | nonsynonymous SNV |
| chr12 | 50748374 | C | A | FAM186A | nonsynonymous SNV |
| chr12 | 52309017 | G | A | ACVRL1 | nonsynonymous SNV |
| chr12 | 54970377 | C | G | PDE1B | nonsynonymous SNV |
| chr12 | 70980858 | C | T | PTPRB | nonsynonymous SNV |
| chr12 | 95927897 | G | T | USP44 | nonsynonymous SNV |
| chr12 | 105199049 | C | T | SLC41A2 | nonsynonymous SNV |
| chr14 | 58917299 | C | T | KIAA0586 | nonsynonymous SNV |
| chr14 | 77915644 | C | T | VIPAS39 | nonsynonymous SNV |
| chr14 | 77951213 | G | C | ISM2 | nonsynonymous SNV |
| chr14 | 91782107 | C | T | CCDC88C | nonsynonymous SNV |
| chr14 | 105411351 | C | G | AHNAK2 | nonsynonymous SNV |
| chr15 | 33445825 | C | A | FMN1 | nonsynonymous SNV |
| chr15 | 40628838 | G | A | C15orf52 | nonsynonymous SNV |
| chr15 | 42133274 | G | A | JMJD7-PLA2G4B,PLA2G4B | nonsynonymous SNV |
| chr15 | 89398221 | C | T | ACAN | nonsynonymous SNV |
| chr16 | 602769 | G | A | CAPN15 | nonsynonymous SNV |
| chr16 | 2160755 | G | C | PKD1 | nonsynonymous SNV |
| chr16 | 9010933 | T | A | USP7 | nonsynonymous SNV |
| chr16 | 10867265 | C | T | TVP23A | nonsynonymous SNV |
| chr16 | 14355082 | C | A | MKL2 | nonsynonymous SNV |
| chr16 | 20441015 | C | A | ACSM5 | nonsynonymous SNV |
| chr16 | 31154138 | G | A | PRSS36 | nonsynonymous SNV |
| chr16 | 56545189 | T | A | BBS2 | nonsynonymous SNV |
| chr16 | 67314207 | T | C | PLEKHG4 | nonsynonymous SNV |
| chr16 | 72863710 | G | A | ZFHX3 | nonsynonymous SNV |
| chr16 | 72863711 | G | A | ZFHX3 | nonsynonymous SNV |
| chr17 | 263779 | C | T | C17orf97 | nonsynonymous SNV |
| chr17 | 3181644 | G | T | OR3A2 | nonsynonymous SNV |
| chr17 | 4045841 | C | G | ZZEF1 | nonsynonymous SNV |
| chr17 | 6945876 | G | A | SLC16A11 | nonsynonymous SNV |
| chr17 | 9729493 | C | T | GLP2R | nonsynonymous SNV |
| chr17 | 10546254 | G | C | MYH3 | nonsynonymous SNV |
| chr17 | 12893351 | C | G | ARHGAP44 | nonsynonymous SNV |
| chr17 | 26947547 | G | A | KIAA0100 | nonsynonymous SNV |
| chr17 | 42092194 | C | T | TMEM101 | nonsynonymous SNV |
| chr17 | 48202304 | C | T | SAMD14 | nonsynonymous SNV |
| chr18 | 2688415 | C | A | SMCHD1 | nonsynonymous SNV |
| chr18 | 3215191 | G | T | MYOM1 | nonsynonymous SNV |
| chr18 | 31803000 | C | T | NOL4 | nonsynonymous SNV |
| chr18 | 33694293 | T | C | SLC39A6 | nonsynonymous SNV |
| chr18 | 72775166 | C | A | ZNF407 | nonsynonymous SNV |
| chr19 | 622199 | G | A | POLRMT | nonsynonymous SNV |
| chr19 | 1220644 | C | T | STK11 | nonsynonymous SNV |
| chr19 | 7170656 | G | A | INSR | nonsynonymous SNV |
| chr19 | 19414195 | C | G | SUGP1 | nonsynonymous SNV |
| chr19 | 34262993 | G | C | CHST8 | nonsynonymous SNV |
| chr19 | 43688966 | C | G | PSG5 | nonsynonymous SNV |
| chr19 | 49136874 | C | G | DBP | nonsynonymous SNV |
| chr19 | 49621203 | T | A | LIN7B | nonsynonymous SNV |
| chr19 | 49934318 | G | C | SLC17A7 | nonsynonymous SNV |
| chr19 | 50156692 | G | T | SCAF1 | nonsynonymous SNV |
| chr19 | 54802513 | G | A | LILRA3 | nonsynonymous SNV |
| chr19 | 56369393 | C | T | NLRP4 | nonsynonymous SNV |
| chr19 | 56410206 | C | T | NLRP13 | nonsynonymous SNV |
| chr20 | 30556385 | G | C | XKR7 | nonsynonymous SNV |
| chr20 | 40770612 | C | T | PTPRT | nonsynonymous SNV |
| chr20 | 60887030 | G | A | LAMA5 | nonsynonymous SNV |
| chr20 | 62326914 | C | A | RTEL1 | nonsynonymous SNV |
| chr21 | 37603036 | G | A | DOPEY2 | nonsynonymous SNV |
| chr22 | 21975914 | G | A | UBE2L3 | nonsynonymous SNV |
| chr22 | 31090202 | G | A | OSBP2 | nonsynonymous SNV |
| chr22 | 40391481 | G | C | FAM83F | nonsynonymous SNV |
| chr22 | 40661358 | A | T | TNRC6B | nonsynonymous SNV |
| chr22 | 41936714 | G | A | POLR3H | nonsynonymous SNV |
| chr22 | 50716589 | T | A | PLXNB2 | nonsynonymous SNV |
| chrX | 70342960 | G | A | MED12 | nonsynonymous SNV |
| chrX | 108647580 | G | C | GUCY2F | nonsynonymous SNV |
| chrX | 114419092 | A | G | LRCH2 | nonsynonymous SNV |
| chr2 | 88883039 | G | A | EIF2AK3 | stopgain |
| chr2 | 191125947 | G | C | HIBCH | stopgain |
| chr3 | 172165877 | C | T | GHSR | stopgain |
| chr5 | 179707537 | G | A | MAPK9 | stopgain |
| chr9 | 140509155 | C | T | ARRDC1 | stopgain |
| chr10 | 86273580 | A | T | CCSER2 | stopgain |
| chr11 | 57995525 | C | A | OR10Q1 | stopgain |
| chr12 | 56109851 | C | T | BLOC1S1 | stopgain |
| chr16 | 89971393 | C | G | TCF25 | stopgain |
| chr19 | 6471308 | G | A | DENND1C | stopgain |
