# Supplementary material for: Adaptive resistance to PI3Kα-selective inhibitor CYH33 is mediated by genomic and transcriptomic alterations in ESCC cells
Source: Cell Death Dis. 2021 Jan 14;12(1):85. doi: 10.1038/s41419-020-03370-4 (PMC7809409; doi:10.1038/s41419-020-03370-4)
Supplement: Supplementary file 7 — Table S5 [file 41419_2020_3370_MOESM7_ESM.docx]

|  |  |  |  | KYSE180C vs KYSE180 |
| --- | --- | --- | --- | --- |
| Chrom | Start | End | Name | Haploid |
| chr8 | 123793700 | 123986955 | ZHX2 | 2.61 |
| chr8 | 124025203 | 124054863 | DERL1 | 2.53 |
| chr8 | 124084719 | 124164592 | TBC1D31 | 2.44 |
| chr8 | 124191086 | 124222519 | FAM83A | 2.69 |
| chr8 | 124213211 | 124215183 | FAM83A-AS1 | 2.7 |
| chr8 | 124227827 | 124228303 | MIR4663 | 2.46 |
| chr8 | 124231995 | 124253838 | C8orf76 | 2.54 |
| chr8 | 124238228 | 124286927 | ZHX1-C8orf76 | 2.47 |
| chr8 | 124260489 | 124287981 | ZHX1 | 2.42 |
| chr8 | 124331890 | 124408905 | ATAD2 | 2.45 |
| chr8 | 124428764 | 124454680 | WDYHV1 | 2.57 |
| chr8 | 124509926 | 124553693 | FBXO32 | 2.57 |
| chr8 | 124657714 | 124665390 | KLHL38 | 2.47 |
| chr8 | 124692833 | 124749847 | ANXA13 | 2.5 |
| chr8 | 124780681 | 124827890 | FAM91A1 | 2.41 |
| chr8 | 124864026 | 125132502 | FER1L6 | 2.5 |
| chr8 | 124996177 | 125053223 | FER1L6-AS1 | 2.49 |
| chr8 | 125058111 | 125183963 | FER1L6-AS2 | 2.48 |
| chr8 | 125322958 | 125385140 | TMEM65 | 2.43 |
| chr8 | 125462847 | 125465466 | TRMT12 | 2.73 |
| chr8 | 125474537 | 125487004 | RNF139-AS1 | 2.59 |
| chr8 | 125486807 | 125501059 | RNF139 | 2.48 |
| chr8 | 125500534 | 125551529 | TATDN1 | 2.51 |
| chr8 | 125520555 | 125521017 | MIR6844 | 2.45 |
| chr8 | 125551142 | 125562427 | NDUFB9 | 2.51 |
| chr8 | 125562810 | 125740948 | MTSS1 | 2.59 |
| chr8 | 125954049 | 125963537 | LINC00964 | 2.52 |
| chr8 | 125985338 | 125991830 | ZNF572 | 2.42 |
| chr8 | 126010519 | 126034725 | SQLE | 2.46 |
| chr8 | 126036302 | 126104261 | KIAA0196 | 2.5 |
| chr8 | 126103882 | 126379567 | NSMCE2 | 2.52 |
| chr8 | 126442362 | 126450847 | TRIB1 | 2.74 |
| chr8 | 126934566 | 126963641 | LINC00861 | 2.52 |
| chr8 | 127564482 | 127570911 | FAM84B | 3.06 |
| chr8 | 128025198 | 128033459 | PCAT1 | 2.98 |
| chr8 | 128084738 | 128094666 | PCAT2 | 2.93 |
| chr8 | 128091918 | 128105040 | PRNCR1 | 2.87 |
| chr8 | 128199830 | 128210072 | LINC01245 | 2.87 |
| chr8 | 128219426 | 128231713 | CCAT1 | 2.95 |
| chr8 | 128256681 | 128405076 | CASC21 | 2.9 |
| chr8 | 128301720 | 128494584 | CASC8 | 2.92 |
| chr8 | 128412443 | 128414595 | CCAT2 | 3.1 |
| chr8 | 128427656 | 128429641 | POU5F1B | 2.86 |
| chr8 | 128712652 | 128746413 | CASC11 | 3.07 |
| chr8 | 128748114 | 128753880 | MYC | 3.28 |
| chr8 | 128808007 | 128808474 | MIR1204 | 3.41 |
| chr8 | 128902673 | 129113699 | PVT1 | 3.11 |
| chr8 | 128958604 | 128961169 | TMEM75 | 3.17 |
| chr8 | 128972678 | 128973141 | MIR1205 | 2.73 |
| chr8 | 129020943 | 129021402 | MIR1206 | 2.92 |
| chr8 | 129061197 | 129061684 | MIR1207 | 2.97 |
| chr8 | 129162161 | 129162634 | MIR1208 | 3.48 |
| chr8 | 129417315 | 129577125 | LINC00824 | 2.78 |
| chr8 | 130228512 | 130253686 | LINC00977 | 2.85 |
| chr8 | 130760241 | 130799334 | GSDMC | 2.83 |
| chr8 | 130851638 | 131029097 | FAM49B | 2.77 |
| chr8 | 131020379 | 131020899 | MIR5194 | 2.71 |
| chr8 | 131064150 | 131456106 | ASAP1 | 2.84 |
| chr8 | 131094783 | 131097214 | ASAP1-IT2 | 2.69 |
| chr8 | 131307400 | 131308979 | ASAP1-IT1 | 2.83 |
| chr8 | 131792346 | 132053035 | ADCY8 | 2.8 |
| chr8 | 132916155 | 133026086 | EFR3A | 2.66 |
| chr8 | 133036266 | 133071827 | OC90 | 2.98 |
| chr8 | 133073532 | 133117712 | HHLA1 | 3.1 |
| chr8 | 133132904 | 133493204 | KCNQ3 | 3.18 |
