# Supplementary material for: Adaptive resistance to PI3Kα-selective inhibitor CYH33 is mediated by genomic and transcriptomic alterations in ESCC cells
Source: Cell Death Dis. 2021 Jan 14;12(1):85. doi: 10.1038/s41419-020-03370-4 (PMC7809409; doi:10.1038/s41419-020-03370-4)
Supplement: Supplementary file 8 — Table S6 [file 41419_2020_3370_MOESM8_ESM.docx]

|  |  |  |  | KYSE410C vs KYSE410 |
| --- | --- | --- | --- | --- |
| Chrom | Start | End | Name | Haploid |
| chr22 | 18893535 | 18899801 | DGCR6 | 2.69 |
| chr22 | 18900086 | 18924266 | PRODH | 2.76 |
| chr22 | 18957810 | 19018955 | DGCR5 | 2.56 |
| chr22 | 19005146 | 19007961 | DGCR9 | 2.71 |
| chr22 | 19009936 | 19011263 | DGCR10 | 2.51 |
| chr22 | 19023594 | 19110167 | DGCR2 | 2.56 |
| chr22 | 19033474 | 19036088 | DGCR11 | 2.39 |
| chr22 | 19117591 | 19132390 | DGCR14 | 2.61 |
| chr22 | 19118120 | 19120336 | TSSK2 | 2.98 |
| chr22 | 19136303 | 19137996 | GSC2 | 2.56 |
| chr22 | 19159018 | 19160545 | LINC01311 | 2.85 |
| chr22 | 19162887 | 19166576 | SLC25A1 | 2.66 |
| chr22 | 19166786 | 19279439 | CLTCL1 | 2.48 |
| chr22 | 19318023 | 19419419 | HIRA | 2.48 |
| chr22 | 19419835 | 19423796 | MRPL40 | 2.63 |
| chr22 | 19428209 | 19435955 | C22orf39 | 2.58 |
| chr22 | 19437263 | 19466938 | UFD1L | 2.48 |
| chr22 | 19467148 | 19508335 | CDC45 | 2.5 |
| chr22 | 19510346 | 19513060 | CLDN5 | 3.05 |
| chr22 | 19553452 | 19554562 | LINC00895 | 2.9 |
| chr22 | 19701786 | 19711045 | SEPT5 | 2.81 |
| chr22 | 19704542 | 19712497 | SEPT5-GP1BB | 2.69 |
| chr22 | 19710865 | 19712497 | GP1BB | 2.63 |
| chr22 | 19744025 | 19771312 | TBX1 | 2.51 |
| chr22 | 19775733 | 19842662 | GNB1L | 2.64 |
| chr22 | 19833460 | 19842571 | C22orf29 | 2.7 |
| chr22 | 19862840 | 19929715 | TXNRD2 | 2.65 |
| chr22 | 19929062 | 19957698 | COMT | 2.55 |
| chr22 | 19951075 | 19951557 | MIR4761 | 2.59 |
| chr22 | 19957201 | 20004509 | ARVCF | 2.67 |
| chr22 | 20004322 | 20053649 | TANGO2 | 2.69 |
| chr22 | 20020461 | 20020943 | MIR185 | 3.26 |
| chr22 | 20067554 | 20099600 | DGCR8 | 2.51 |
| chr22 | 20073068 | 20073556 | MIR3618 | 2.69 |
| chr22 | 20073380 | 20073865 | MIR1306 | 2.8 |
| chr22 | 20099188 | 20105018 | TRMT2A | 2.87 |
| chr22 | 20102008 | 20102474 | MIR6816 | 2.51 |
| chr22 | 20103260 | 20115080 | RANBP1 | 2.59 |
| chr22 | 20119163 | 20135730 | ZDHHC8 | 2.78 |
| chr22 | 20135908 | 20137631 | LOC388849 | 2.83 |
| chr22 | 20186052 | 20192641 | LOC284865 | 2.7 |
| chr22 | 20193654 | 20196260 | LINC00896 | 2.64 |
| chr22 | 20228737 | 20256016 | RTN4R | 3.35 |
| chr22 | 20236456 | 20236934 | MIR1286 | 3.8 |
| chr22 | 20301560 | 20307828 | DGCR6L | 3.44 |
| chr22 | 20377468 | 20380640 | TMEM191B | 2.18 |
| chr22 | 20383530 | 20398895 | PI4KAP1 | 2.42 |
| chr22 | 20455793 | 20461986 | RIMBP3 | 3.11 |
| chr22 | 20748204 | 20762953 | ZNF74 | 2.96 |
| chr22 | 20778673 | 20792346 | SCARF2 | 3.23 |
| chr22 | 20795605 | 20850370 | KLHL22 | 2.96 |
| chr22 | 20861628 | 20942119 | MED15 | 3.06 |
| chr22 | 21043642 | 21046209 | POM121L4P | 3.51 |
| chr22 | 21055201 | 21059091 | TMEM191A | 3.41 |
| chr22 | 21061778 | 21213300 | PI4KA | 2.99 |
| chr22 | 21128182 | 21142208 | SERPIND1 | 3.03 |
| chr22 | 21213091 | 21245701 | SNAP29 | 2.89 |
| chr22 | 21271513 | 21308237 | CRKL | 2.94 |
| chr22 | 21311179 | 21319168 | LOC101928891 | 3.04 |
| chr22 | 21319217 | 21335849 | AIFM3 | 3.12 |
| chr22 | 21336357 | 21353526 | LZTR1 | 3.18 |
| chr22 | 21353860 | 21356604 | THAP7 | 3.5 |
| chr22 | 21356010 | 21364863 | THAP7-AS1 | 2.97 |
| chr22 | 21362295 | 21368776 | TUBA3FP | 3.19 |
| chr22 | 21369241 | 21382502 | P2RX6 | 3.14 |
| chr22 | 21382806 | 21387047 | SLC7A4 | 3.16 |
| chr22 | 21396480 | 21398738 | P2RX6P | 2.96 |
| chr22 | 21400048 | 21418657 | LOC400891 | 3.01 |
| chr22 | 21457104 | 21476775 | BCRP2 | 2.89 |
| chr22 | 21520991 | 21546645 | FAM230B | 2.79 |
| chr22 | 21636513 | 21652215 | POM121L8P | 2.94 |
| chr22 | 21737462 | 21743655 | RIMBP3C | 2.97 |
| chr22 | 21737839 | 21743655 | RIMBP3B | 3.02 |
| chr22 | 21771492 | 21805950 | HIC2 | 2.02 |
| chr22 | 21821258 | 21824424 | TMEM191C | 2.55 |
| chr22 | 21827086 | 21871980 | PI4KAP2 | 2.06 |
| chr22 | 21899757 | 21905950 | RIMBP3C | 2.67 |
| chr22 | 21899757 | 21905573 | RIMBP3B | 2.66 |
