# Supplementary material for: Adaptive resistance to PI3Kα-selective inhibitor CYH33 is mediated by genomic and transcriptomic alterations in ESCC cells
Source: Cell Death Dis. 2021 Jan 14;12(1):85. doi: 10.1038/s41419-020-03370-4 (PMC7809409; doi:10.1038/s41419-020-03370-4)
Supplement: Supplementary file 9 — Table S7 [file 41419_2020_3370_MOESM9_ESM.docx]

|  |  |  |  | KYSE510C vs KYSE510 |
| --- | --- | --- | --- | --- |
| Chrom | Start | End | Name | Haploid |
| chr2 | 175936777 | 176033134 | ATF2 | 3.15 |
| chr2 | 176032160 | 176032637 | MIR933 | 3.46 |
| chr2 | 176040785 | 176046690 | ATP5G3 | 3.42 |
| chr2 | 176790209 | 176867218 | KIAA1715 | 3.77 |
| chr2 | 176944634 | 176948890 | EVX2 | 4.44 |
| chr2 | 176957331 | 176960866 | HOXD13 | 4.42 |
| chr2 | 176964329 | 176965688 | HOXD12 | 3.9 |
| chr2 | 176971883 | 176974516 | HOXD11 | 3.79 |
| chr2 | 176981291 | 176984870 | HOXD10 | 4.2 |
| chr2 | 176987212 | 176989845 | HOXD9 | 4.25 |
| chr2 | 176994221 | 176997623 | HOXD8 | 4.65 |
| chr2 | 176999368 | 177002026 | HOXD-AS2 | 4.65 |
| chr2 | 177014830 | 177015340 | MIR10B | 4.76 |
| chr2 | 177015912 | 177018149 | HOXD4 | 4.28 |
| chr2 | 177028604 | 177038026 | HOXD3 | 4.28 |
| chr2 | 177037716 | 177053886 | HAGLR | 4.3 |
| chr2 | 177042693 | 177043937 | HAGLROS | 4.13 |
| chr2 | 177053106 | 177055835 | HOXD1 | 3.97 |
| chr2 | 177133922 | 177202953 | MTX2 | 4.39 |
| chr2 | 177465507 | 177465980 | MIR1246 | 5.37 |
| chr2 | 177494108 | 177502502 | LINC01116 | 4.47 |
| chr2 | 177502281 | 177520886 | LOC102724224 | 4.54 |
| chr2 | 178077221 | 178088885 | HNRNPA3 | 5.2 |
| chr2 | 178077253 | 178077726 | MIR4444-2 | 4.61 |
| chr2 | 178077253 | 178077726 | MIR4444-1 | 4.61 |
| chr2 | 178094830 | 178130059 | NFE2L2 | 6.88 |
| chr2 | 178120472 | 178120938 | MIR3128 | 7.94 |
| chr2 | 178148035 | 178257619 | LOC100130691 | 6.64 |
| chr2 | 178178333 | 178178810 | MIR6512 | 7.19 |
| chr2 | 178257270 | 178408764 | AGPS | 6.38 |
| chr2 | 178414680 | 178417724 | TTC30B | 7.09 |
| chr2 | 178478825 | 178483894 | TTC30A | 6.67 |
| chr2 | 178487776 | 178973266 | PDE11A | 5.34 |
| chr2 | 178976981 | 178994582 | RBM45 | 5.39 |
| chr2 | 179059007 | 179264360 | OSBPL6 | 5.37 |
| chr2 | 179246604 | 179541209 | MIR548N | 5.41 |
| chr2 | 179278185 | 179305170 | LOC101927027 | 5.49 |
| chr2 | 179295940 | 179316158 | PRKRA | 5.47 |
| chr2 | 179315962 | 179326310 | DFNB59 | 5.65 |
| chr2 | 179328190 | 179343555 | FKBP7 | 5.45 |
| chr2 | 179344998 | 179369982 | PLEKHA3 | 5.6 |
| chr2 | 179387353 | 179485144 | TTN-AS1 | 5.41 |
| chr2 | 179390516 | 179672350 | TTN | 5.28 |
| chr2 | 179641452 | 179644890 | LOC101927055 | 5.3 |
| chr2 | 179694283 | 179914986 | CCDC141 | 5.14 |
| chr2 | 179966218 | 180129550 | SESTD1 | 3.61 |
| chr2 | 180306510 | 180726432 | ZNF385B | 2.76 |
| chr2 | 180725362 | 180725835 | MIR1258 | 2.01 |
| chr2 | 180809403 | 180871980 | CWC22 | 2.74 |
| chr2 | 181556630 | 181781866 | SCHLAP1 | 2.73 |
| chr2 | 181844911 | 181928354 | UBE2E3 | 2.82 |
| chr2 | 182170119 | 182170579 | MIR4437 | 3.07 |
| chr2 | 182321418 | 182402668 | ITGA4 | 3.02 |
| chr2 | 182401200 | 182522034 | CERKL | 3.1 |
| chr2 | 182540632 | 182545592 | NEUROD1 | 3.24 |
| chr2 | 182756242 | 182795664 | SSFA2 | 2.87 |
| chr2 | 182818767 | 182996309 | PPP1R1C | 2.94 |
| chr2 | 183004561 | 183387772 | PDE1A | 2.83 |
| chr2 | 183580567 | 183644950 | DNAJC10 | 2.91 |
| chr2 | 183697804 | 183731698 | FRZB | 3.06 |
| chr2 | 183789378 | 183903786 | NCKAP1 | 2.97 |
| chr2 | 183943086 | 183964922 | DUSP19 | 3.29 |
| chr2 | 183982017 | 184026612 | NUP35 | 3.32 |
| chr2 | 185462892 | 185804414 | ZNF804A | 2.88 |
| chr2 | 186584400 | 186605404 | LOC101927196 | 2.75 |
| chr2 | 186603154 | 186698216 | FSIP2 | 2.65 |
| chr2 | 186898060 | 186951244 | LOC101927217 | 2.52 |
| chr2 | 187350684 | 187374287 | ZC3H15 | 2.68 |
| chr2 | 187454589 | 187545829 | ITGAV | 2.68 |
| chr2 | 187558588 | 187628712 | FAM171B | 2.75 |
| chr2 | 187692006 | 187714097 | ZSWIM2 | 2.62 |
| chr2 | 188206489 | 188313221 | CALCRL | 2.68 |
| chr2 | 188328757 | 188419419 | TFPI | 2.76 |
| chr2 | 189156195 | 189460852 | GULP1 | 2.59 |
| chr2 | 189162018 | 189162515 | MIR561 | 2.22 |
| chr2 | 189598264 | 189655031 | DIRC1 | 2.62 |
| chr2 | 189838898 | 189877672 | COL3A1 | 2.72 |
| chr2 | 189842617 | 189843086 | MIR1245A | 2.42 |
| chr2 | 189842619 | 189843087 | MIR1245B | 2.42 |
| chr2 | 189860155 | 189860618 | MIR3606 | 2.32 |
| chr2 | 189896440 | 190044805 | COL5A2 | 2.7 |
| chr2 | 189997561 | 189998037 | MIR3129 | 2.45 |
| chr2 | 190305958 | 190340464 | WDR75 | 2.69 |
| chr2 | 190425115 | 190445737 | SLC40A1 | 2.82 |
| chr2 | 190525924 | 190535757 | ASNSD1 | 2.79 |
| chr2 | 190540510 | 190611576 | ANKAR | 2.72 |
| chr2 | 190611185 | 190628124 | OSGEPL1 | 2.6 |
| chr2 | 190627305 | 190630482 | OSGEPL1-AS1 | 2.87 |
| chr2 | 190634792 | 190649297 | ORMDL1 | 2.68 |
| chr2 | 190648610 | 190742555 | PMS1 | 2.74 |
